# Construction of a Stable Lanthanide Metal-Organic Framework as a Luminescent Probe for Rapid Naked-Eye Recognition of Fe^3+^ and Acetone

**DOI:** 10.3390/molecules26061695

**Published:** 2021-03-18

**Authors:** Jiayishuo Wang, Muxin Yu, Lian Chen, Zhijia Li, Shengchang Li, Feilong Jiang, Maochun Hong

**Affiliations:** 1 Key Laboratory of Optoelectronic Materials Chemistry and Physics, Fujian Institute of Research on the Structure of Matter, Chinese Academy of Sciences, Fuzhou 350002, China; wangjiayishuo@fjirsm.ac.cn (J.W.); muxinyu@fjirsm.ac.cn (M.Y.); lizhijia@fjirsm.ac.cn (Z.L.); lishengchang@fjirsm.ac.cn (S.L.); fjiang@fjirsm.ac.cn (F.J.); 2 University of the Chinese Academy of Sciences, Beijing 100049, China; 3 Organic Optoelectronics Engineering Research Centre of Fujian’s Universities, College of Electronics and Information Science, Fujian Jiangxia University, Fuzhou 350108, China

**Keywords:** metal-organic frameworks, lanthanides, luminescence, Fe^3+^ sensors, acetone sensors

## Abstract

Four lanthanide metal-organic frameworks (Ln-MOFs), namely {[Me_2_NH_2_][LnL]·2H_2_O}_n_ (Ln = Eu **1**, Tb **2**, Dy **3**, Gd **4**), have been constructed from a new tetradentate ligand 1-(3,5-dicarboxylatobenzyl)-3,5-pyrazole dicarboxylic acid (H_4_L). These isostructural Ln-MOFs, crystallizing in the monoclinic *P*2_1_/c space group, feature a 3D structure with 7.5 Å × 9.8 Å channels along the *b* axis and the point symbol of {4^10^.6^14^.8^4^} {4^5^.6}_2_. The framework shows high air and hydrolytic stability, which can keep stable after exposed to humid air for 30 days or immersed in water for seven days. Four MOFs with different lanthanide ions (Eu^3+^, Tb^3+^, Dy^3+^, and Gd^3+^) ions exhibit red, green, yellow, and blue emissions, respectively. The Tb-MOF emitting bright green luminescence can selectively and rapidly (<40 s) detect Fe^3+^ in aqueous media via a fluorescence quenching effect. The detection shows excellent anti-inference ability toward many other cations and can be easily recognized by naked eyes. In addition, it can also be utilized as a rapid fluorescent sensor to detect acetone solvent as well as acetone vapor. Similar results of sensing experiments were observed from Eu-MOF. The sensing mechanism are further discussed.

## 1. Introduction

Environmental and ecological problems have caused widespread concern of scientists in recent years. As an essential metal element in human metabolisms, Fe^3+^ ion plays a crucial role in muscle function, brain function, and hemoglobin [1,2]. Serious problems can be caused by the overloading or deficiency in the Fe^3+^ ion, such as immunosuppression, cognitive decline, and iron deficient anemia [3]. Likewise, acetone (CH_3_COCH_3_), which is one of the critical members of volatile organic solvents (VOCs), has experienced an ever-growing attention not only in the laboratory and industry but also in the household, since it can cause irreversible damage to the human body, such as inhibiting breathing and causing dyspnea [4]. Accordingly, rapid and convenient detection of Fe^3+^ and acetone are increasingly essential for the environmental and ecological system.

**Scheme 1:**
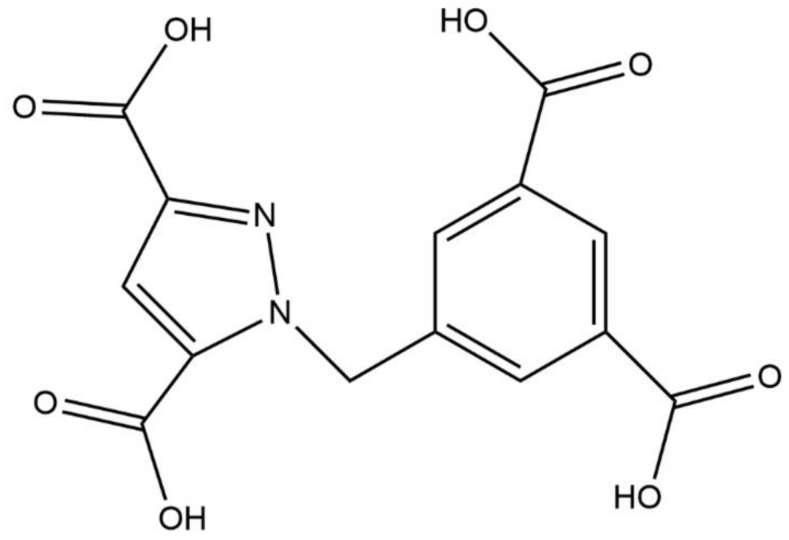
1-(3,5-dicarboxylatobenzyl)-3,5-pyrazole dicarboxylic acid (H_4_L).

In the recent decades, metal-organic frameworks (MOFs) have drawn increasing research interest on account of their unique features in their structures, such as permanent porosity, tunable structures, functions, and so on [5,6,7,8]. The potential applications of MOFs include but not limit to catalysis [9,10,11], sensing [12,13,14,15,16,17,18,19,20], optics [21,22,23,24,25], gas storage and separation [26,27,28,29], bio-imaging, and drug delivery [30,31,32,33]. In particular, lanthanide metal-organic frameworks (Ln-MOFs), displaying bright luminescence with large Stokes shifts, high quantum yields, and long lifetimes, are widely used as versatile materials in optics and sensing [34]. The forerunners have made great efforts on the recognition of environmentally and biologically relative species based on luminescent Ln-MOFs [35,36]. However, most of the MOFs reported for luminescent sensing are applied in nonaqueous media because of their poor hydrolytic stabilities [37]. In addition, the reports of rapid detection are few. The poor stabilities of the structures and the time consumption in the detection limit the further applications of MOFs in luminescent sensing. In light of these facts, we attempt to construct multifunctional Ln-MOFs for sensing in aqueous media conveniently and rapidly. Herein, a tetracarboxylic acid ligand, 1-(3,5-dicarboxylatobenzyl)-3,5-pyrazole dicarboxylic acid (H_4_L, Scheme 1), which can provide versatile coordination modes and Lewis base sites, was employed to construct four novel MOFs {[Me_2_NH_2_][LnL]·2H_2_O}_n_ (Ln = Eu **1**, Tb **2**, Dy **3**, and Gd **4**). The MOFs demonstrate excellent air and water stabilities as well as colorful luminescence. Based on these virtues, a convenient and fast-response luminescent sensor toward the Fe^3+^ ion in aqueous solution with good anti-inference ability was fabricated. It also shows the potential for quickly sensing acetone solvent as well as its vapor in the air. The simplicity, visualization, and high time efficiency of this method make it a competitive dual-functional fluorescent probe.

## 2. Results and Discussion

### 2.1. Structure Description of {[Me_2_NH_2_][LnL]·2H_2_O}_n_ (Ln = Eu ***1***, Tb ***2***, Dy ***3***, Gd ***4***) (Compounds ***1***–***4***)

Crystal data and refinement results for compounds **1–4** are listed in Table 1.

**Figure 1:**
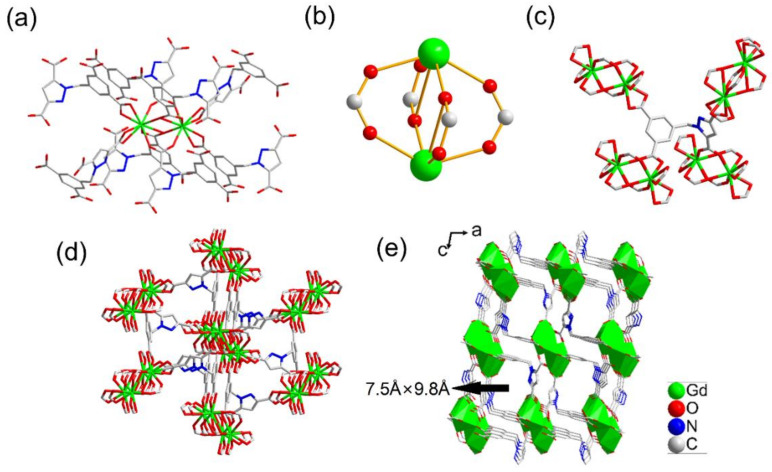
(**a**) Coordination environment of the Gd^3+^ ion in Gd-metal-organic frameworks (MOF) (Gd, green, O, red, N, blue, C, gray). (**b**) The binuclear metal cluster unit [Gd_2_(COO)_4_]. (**c**) The coordination mode of the [L]^4−^ ligand. (**d**) The coordination mode of the binuclear metal cluster unit [Gd_2_(COO)_4_]. (**e**) The 3D framework viewed along the b-axis.

Single crystal X-ray diffraction analysis (SCXRD) shows that the four compounds are isomorphic. Therefore, we choose **4** (Gd-MOF) as a representative to elaborate the structure. Crystal structure analysis reveals that it crystallizes in space group P2_1_/c. The asymmetric unit contains a crystallographically-independent Gd^3+^ ion, a fully deprotonated [L]^4−^ ligand, and an isolated dimethylamine cation (Figure S2). In the compound, the coordination environment of the nine-coordinated gadolinium center is composed of six oxygen atoms from three chelating bidentate carboxylate groups and three oxygen atoms from three μ_2_-bridging carboxylate groups (Figure 1a). The Gd1 atom and its corresponding symmetrically generated one are bridged by four bidentate carboxylic groups of four [L]^4−^ ligands to form a binuclear metal cluster unit [Gd_2_(COO)_4_] (Figure 1b). The coordination mode of the ligand is a μ_6_-bridging mode connecting with six Gd^3+^ ions from four [Gd_2_(COO)_4_] clusters, where two carboxylate groups adopt a chelating bidentate mode and the other two carboxylate groups adopt a μ_2_-bridging bidentate mode, respectively (Figure 1c and Figure S3). Each [Gd_2_(COO)_4_] cluster is connected with the other 14 binuclear clusters through eight [L]^4−^ ligands (Figure 1d). As shown in Figure 1e, the binuclear metal cluster units are further interconnected to form an infinite 3D framework through the organic linkers, showing a 1D open channel along the b-axis with the approximate size of 7.5 Å × 9.8 Å.

**Figure 2:**
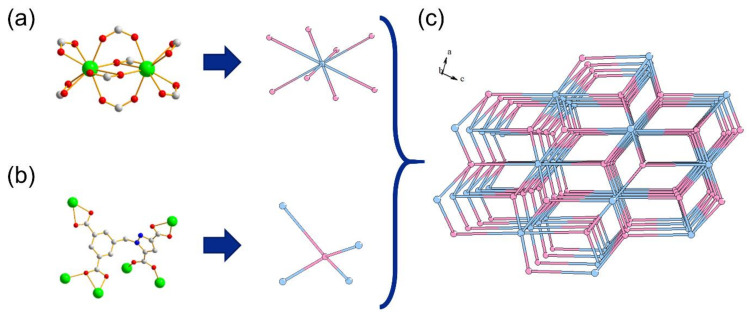
(**a**) The simplified eight-connected node of the binuclear unit. (**b**) The simplified 4-connected linker. (**c**) The (4, 8)-connected topological 3D net with the Schläfli symbol {4^10^.6^14^.8^4^} {4^5^.6}_2_.

Topological analysis [38] shows that the structure can be simplified as a new (4, 8)-connected net with stoichiometry (4−c) (8−c)_2_ and point symbol of {4^10^.6^14^.8^4^} {4^5^.6}_2_ when the binuclear metal cluster units and ligands are considered as eight-connected nodes and four-connected linkers, respectively (Figure 2). Powder X-ray diffraction (PXRD) patterns were then recorded to analyze the phase purity of the obtained compounds. The results reveal that the samples of four MOFs are of high purity (Figure S4).

### 2.2. Framework Stability

The framework stability of MOFs is of vital importance for their practical applications. Therefore, the following experiments were carried out. First, thermogravimetry analyses (TGA) for the four compounds were studied (Figure S5). The results show that the thermogravimetric curves of the four samples overlap together. When the temperature rises to 180 °C, the weight loss is about 6.3%, which should be the escape of two water molecules. The frameworks keep stable up to 288 °C and begin to collapse when the temperature exceeds 288 °C. Since the MOFs have the same frameworks, **2** is selected for the further tests. A thermo-diffractogram obtained from 30 °C to 450 °C demonstrates the crystalline structure keeps stable from 30–200 °C, slightly changes at 300 °C, and finally collapses when the temperature comes up to 400 °C (Figure S6a). The observation is in accordance with the result from TGA. The air stability is determined by collecting PXRD patterns after a period of exposure in the atmosphere with a humidity of ca. 55%. The unchanged PXRD patterns reveal that the framework can survive in the humid air for more than 30 days (Figure S6b). The PXRD patterns of the samples immersed in water shows that the water stability of the framework is excellent (remain stable for more than 7 days (Figure S6c)). Chemical stability of the MOF was also evaluated. The samples were immersed in aqueous solutions with the pH values varying from 2 to 11 for 42 h. Then, the filtered samples were dried in air. The nearly unchanged PXRD patterns with pH values from 5 to 10 indicate relatively good chemical stability (Figure S6d).

### 2.3. Luminescent Properties

**Figure 3:**
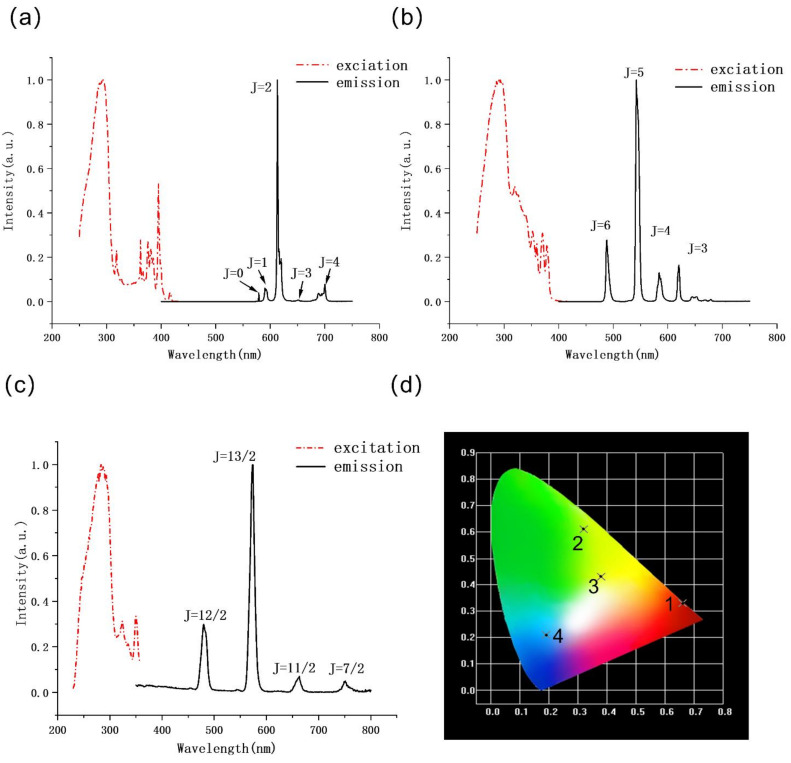
(**a**) The excitation (λ_em_ = 614 nm) and emission spectra (λ_ex_ = 297 nm) of **1**. (**b**) The excitation (λ_em_ = 543 nm) and emission spectra (λ_ex_ = 299 nm) of compound **2**. (**c**) The excitation (λ_em_ = 574 nm) and emission spectra (λ_ex_ = 287 nm) of compound **3**. (**d**) CIE-1931 chromaticity diagram shows the luminescence colors of compounds **1** (0.66, 0.33), **2** (0.32,0.61), **3** (0.38, 0.43), and **4** (0.19, 0.21).

The luminescence properties of the ligand and compounds **1–4** in a solid state are shown in Figure 3 and Figure S7. Ln^3+^ ions suffer from weak light absorption with the absorption coefficients generally less than 10 M^−1^ cm^−1^ due to the “Laporte forbidden” feature of the transitions between the 4f^n^ configurations of the Ln^3+^ Ions. According to the famous “antenna effect” theory founded by Weissman in 1942 [39,40,41], the organic ligand can efficiently absorb light and transfer this energy to the excited states of the central lanthanide ions by overcoming the weak absorption of lanthanide and, thus, leading to the improvement of the emission efficiency. When excited at 297 nm, **1** shows the characteristic red emission with the emissions at 580 nm, 591 nm, 613 nm, 615 nm, and 700 nm corresponding to ^5^D_0_ → ^7^F_J_ (J = 1–4) transitions of Eu^3+^ centers, respectively (Figure 3a). Under the excitation of 299 nm, **2** exhibits characteristic green emission with the peaks at 488 nm, 619 nm, 543 nm, 584 nm, and 619 nm, ascribed to ^5^D_4_ → ^7^F_J_ (J = 6, 5, 4, and 3) transitions of Tb^3+^ ions (Figure 3b). **3** displays yellow emission bands at 479 nm, 573 nm, 663 nm, and 751 nm upon excitation at 287 nm, which are due to ^7^F_9/2_ → ^6^H_J_ (J = 12/2, 13/2, 11/2, 7/2) transitions of Dy^3+^ ions (Figure 3c). Similar bands around 300 nm observed in the excitation spectra (dash lines, Figure 3) of the three complexes may be ascribed to the n→ π* or π→ π* transitions of the ligand. The ligand displays a broad band at 370–550 nm on the excitation of 346 nm (Figure S7a). Apart from Eu^3+^, Tb^3+^, and Dy^3+^ ions, the energy levels of Gd^3+^ ion are too high so that the energy from the lowest triplet state energy level (T1) of the ligand cannot be transferred to the Gd^3+^ center, bringing about the broad blue emission similar to that of the pure ligand (Figure S7b). The phosphorescence spectrum of 4 at 77 K reveals that the triplet state energy level T_1_ of the ligand is 24,570 cm^−1^ (407 nm). The Commission Internationale de l’Éclairage (CIE) 1931 chromaticity diagram shows the different emission colors of the compounds (Figure 3d). The room temperature quantum yields and lifetimes of compounds **1**, **2,** and **3** were also measured and shown in Table S1 and Figure S8. Compared with the low quantum yield of **3**, those of **1** and **2** are as high as 39.29% and 57.77%, respectively, indicating that Eu^3+^ and Tb^3+^ can be better sensitized by the ligand when compared with Dy^3+^. This can be further proven by the larger lifetimes of compounds **1** and **2**.

### 2.4. Sensing of Cations

**Figure 4:**
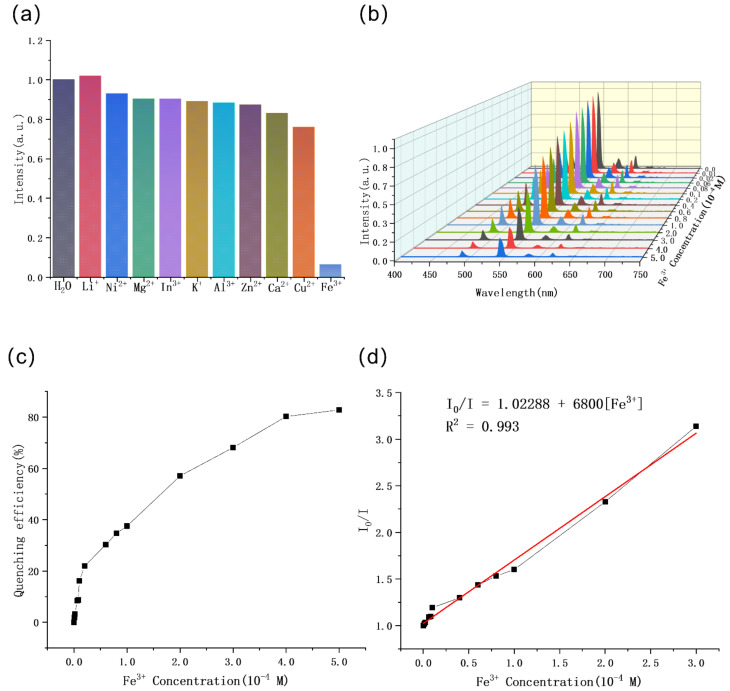
(**a**) Comparison of the ^5^D_4_ → ^7^F_5_ photoluminescence intensity of **2** in water and aqueous solutions containing different metal ions when excited at 299 nm. (**b**) Photoluminescence spectra, (**c**) quenching efficiency, and (**d**) a Stern-Volmer plot of **2** with a gradual increase of the Fe^3+^ concentration in aqueous solution.

Porous characteristics, excellent hydrolytic stability, and intensive luminescence presented above inspire us to explore the potential of the Ln-MOFs for chemical sensing in aqueous media. **2** was selected for cation detection in consideration of its highest luminescent efficiency in four compounds. Then, 3 mg of **2** was dispersed into 3 mL of water, and the fluorescence intensity of the suspension was measured as the blank sample. Then **2** was dispersed into aqueous solution of 1 mM M(NO_3_)_x_ of different kinds of metal ions (M = Li^+^, Ni^2+^, Mg^2+^, In^3+^, K^+^, Al^3+^, Zn^2+^, Ca^2+^, Cu^2+^, or Fe^3+^). The fluorescence spectra of suspensions show that the intensity of 543 nm has slight or negligible changes in the presence of most ions except the Fe^3+^ cation (Figure 4a and Figure S9). A remarkable quenching occurs in the Fe^3+^ ion solution, which can be easily observed under the irradiation of ultraviolet (UV) light with naked eyes. This indicates that **2** can be used to selectively detect the Fe^3+^ ion in aqueous solution.

The concentration-dependent luminescence of Fe^3+^ was studied in the presence of Fe^3+^ from 0 to 5 × 10^−4^ M (Figure 4b). The quenching effect can be clearly observed at the concentration of 6 × 10^−6^ M (Figure 4c,d). The fluorescence quenching efficiency is measured by using the formula: (1 − I/I_0_) × 100%), suggesting that **2** shows a high sensitivity in fluorescence quenching sensing. The quenching effect coefficient (*K*_sv_), which verifies the quenching effect, is calculated according to the Stern–Volmer (SV) equation: I_0_/I = 1 + *K*_sv_ [M], where I_0_ and I represent the luminescent intensities before and after analyte addition, respectively. [M] represents the molar concentration of metal ions. In the concentration from 0 to 3 × 10^−4^ M, the Stern-Volmer quenching curve is close to the first-order linearity, with the correlation coefficient of linear fitting 0.993, and the fitted linear equation I_0_/I = 1.02288 + 6800 [Fe^3+^]. However, there is no linear correlation but only an upward curve when the concentration reaches up to 3 × 10^−4^ M. The non-linear nature suggests the mechanism of the quenching can be attributed to the combination of dynamic and static quenching [42,43].

**Figure 5:**
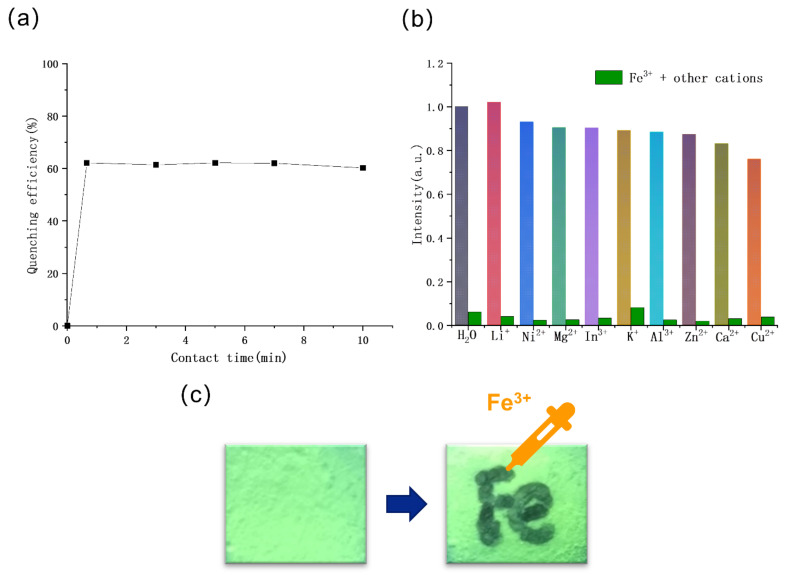
(**a**) Time-dependent quenching efficiency of **2** in the presence of Fe^3+^ solutions (3 × 10^−4^ M). (**b**) Comparison of the ^5^D_4_ → ^7^F_5_ photoluminescence intensity of compound **2** in the pure individual metal ions and mixed metal ions containing 1 mM of Fe^3+^ ion. (**c**) The image of **2** paper-coated sensor treated with 0.01 M Fe^3+^ solution.

During the experiment, it was found that the fluorescence of the sample can be quenched by Fe^3+^ ions in a remarkably short contact time, so we studied the relationship between the Fe^3+^ contact time and the fluorescence intensity. The result showed that the maximum quenching efficiency is achieved in 40 s, and then it remains unchanged (Figure 5a and Figure S10). The phenomenon indicates that the **2** has an ultrafast fluorescence response to the Fe^3+^ ion, which is expected to be applied to real-time sensing.

In order to study the anti-interference ability toward other cations, sensing experiments were carried out in aqueous solution with a mixture of Fe^3+^ ion and other metal cations. The emission intensity of **2** in aqueous solution of mixed cations (Li^+^, Ni^2+^, Mg^2+^, In^3+^, K^+^, Al^3+^, Zn^2+^, Ca^2+^ and Cu^2+^, 1 × 10^−3^ M each) shows only a slight quenching compared with the intensity in pure water (Figure S11). Once the Fe^3+^ ion (1 × 10^−3^ M) was added, the slightly changed fluorescence intensity of **2** in other cationic solutions immediately shows a dramatic decrease (Figure 5b). It indicates that the presence of interference ions has little influence on fluorescence detection of **2** toward the Fe^3+^ ion.

Furthermore, a fluorescent test paper was made to realize a simple, portable, and real-time Fe^3+^ ion sensor. The capillary dipped in 0.01 M Fe^3+^ ion aqueous solution was used to write “Fe” on the fluorescent test paper. The position with writing marks was quenched immediately under the irradiation of a UV lamp (Figure 5c). The above experimental results indicate that the **2** could be employed as a convenient, rapid, and easily-recognized probe for Fe^3+^ with good anti-interference ability.

### 2.5. Sensing of Organic Molecules

**Figure 6:**
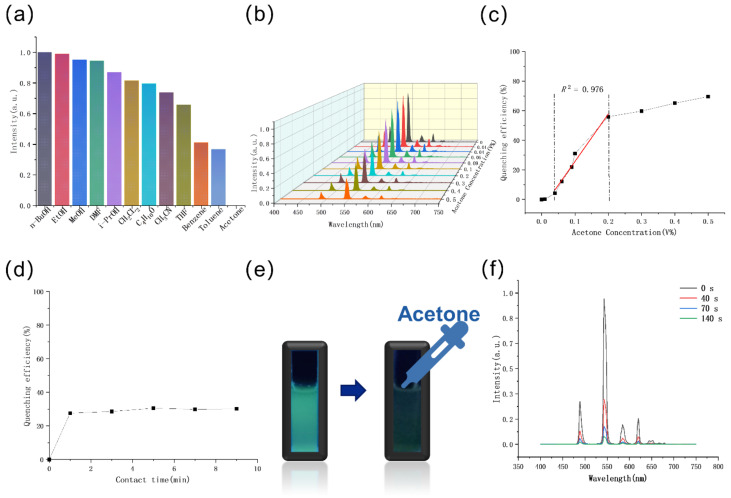
(**a**) Comparison of the ^5^D_4_ → ^7^F_5_ photoluminescence intensity of **2** in different organic molecules when excited at 299 nm. (**b**) Photoluminescence spectra and (**c**) quenching efficiency of **2** with a gradual increase in the concentration of acetone in *N*,*N*′-dimethylformamide (DMF). (**d**) Time-dependent quenching efficiency of **2** suspension in the presence of acetone in DMF (0.1 vol.%). (**e**) Photographs of **2** suspension before and after adding acetone. (**f**) Time-dependent solid photoluminescence spectra of solid-state **2** upon exposure to acetone vapor.

Volatile organic solvents (VOCs) are widely used in laboratory and industrial products. However, they have adverse impact on human health and the environment. As one of the most burgeoning fluorescent probe materials, MOFs are widely used in the detection of organic molecules. To explore the potential application of **2** as a fluorescent sensor for VOCs, a certain amount of its powder was ground and dispersed in different organic solvents, including *n*-BuOH, EtOH, MeOH, *N*,*N*′-dimethylformamide (DMF), *i*-PrOH, CH_2_Cl_2_, C_4_H_10_O, CH_3_CN, tetrahydrofuran (THF), benzene, toluene, *n*-pentane, and acetone. The photoluminescence test was carried out after ultrasonic dispersing the sample evenly. As shown in Figure 6a and Figure S12, the ^5^D_4_ → ^7^F_5_ fluorescence intensity of **2** shows no significant change in most organic solvents while the intensity drops abruptly when **2** is dispersed in acetone. It suggests that **2** may be a selective sensor toward acetone solvent. Then, a concentration-dependent experiment was carried out. Different concentrations of acetone were dissolved in 3 mL of DMF and 3 mg of **2** was then added. The quenching efficiency of the emulsion shows a gradual increase with the growing acetone concentration. A significant variation can be observed when the concentration of acetone is as low as 0.04 vol.%. From 0.04 vol.% to 0.2 vol.%, the increasing trend of the luminescent quenching efficiency of **2** at 543 nm versus the volume ratio of acetone can be fitted with a first-order exponential decay. In addition, the quenching efficiency of **2** toward acetone comes up to 70% at 0.5 vol.% (Figure 6b,c).

The response time of **2** to acetone in liquid was also tested. The result demonstrates that the response of **2** to Fe^3+^ ion has already finished in 40 s (Figure 6d and Figure S13). The phenomenon indicates that the probe responds to acetone very quickly. The apparent brightness change, which can be observed with the naked-eye under the irradiation of the UV light, indicates that **2** can be used to detect acetone conveniently (Figure 6e).

Since acetone is an extremely volatile and highly toxic gas, the detection of acetone vapor is of vital importance. Despite some luminescent sensors for acetone solvent being reported, few efforts have been made for the exploration of the rapid detection of acetone vapor [44,45]. Upon exposure to saturated acetone vapor for 40 s, the fluorescence intensity of solid sample **2** drastically decreases 69.2% (Figure 6f). At 60 s, the decrease comes to 87.8%. Moreover, the decline of the intensity turns to 94.5% when **2** is exposed to acetone for 140 s. These phenomena indicate that compound **2** can detect acetone vapor in an extremely short time.

### 2.6. Sensing Mechanism

The superb detection performance of the compounds prompts us to explore the possible mechanism of luminescence quenching caused by Fe^3+^ and acetone. First, the PXRD patterns of the samples after sensing experiments were recorded (Figures S14 and S15). The nearly unchanged patterns show the samples keep their crystalline structure after treatment, indicating that the luminescence quenching is not caused by the collapse of the framework.

In light of previous studies, competitive energy absorption between analytes and Ln-MOFs may be a very possible reason for the luminescence quenching. With this in mind, the UV-vis spectra of the Fe^3+^ as well as other metal ions (measured in aqueous solution, 2 × 10^−4^ M) and the acetone (measured in DMF solution, 0.2 vol.%) were recorded. As shown in Figure S16, only the UV–vis absorption spectrum of Fe^3+^ has significant absorption around 295 nm, which shows a clear overlap with the excitation spectrum of **2** (Figure 3b). This implies that there is competitive absorption of the light source energy between Fe^3+^ and **2**, and, hence, leads to the quenching behavior. Similarly, spectral overlap is observed between the excitation spectrum of compound **2** and the UV-vis absorption spectrum of acetone ranging from 260 nm to 323 nm (Figure S17).

Based on the above results, we surmise that the main reason of fluorescence quenching can be attributed to the competitive energy absorption between analytes (Fe^3+^ and acetone) and Ln-MOFs. However, we still wonder if there are any other possible mechanisms involved in the luminescence quenching. UV-vis adsorption spectra of Fe^3+^ aqueous solution with different concentrations were then recorded. As Figure S18 shows, there is almost no absorption near 300 nm when the concentration of Fe^3+^ ions is lowered to 6 × 10^−6^ M, but still ca. 8.5% luminescence quenching occurs. This indicates that there may be another reason for the quenching. Furthermore, it is worth noting that **2** shows clear fluorescence quenching towards acetone vapor. In this sensing experiment, luminescence of solid samples instead of suspension was recorded. Thus, the influence caused by the absorption of acetone can be nearly neglected. These facts give us a clue that there may be another mechanism in the quenching process.

To further investigate the other possible mechanism, the isomorphic **1** was chosen to implement the same sensing tests of metal ions and organic solvents. The outcomes of the experiments are consistent with those of **2** (Figures S19 and S20). The luminescence of Eu-MOF also shows selectivity responses towards Fe^3+^ and acetone. The results give a reasonable indication that the other sensing mechanism does not come from the Tb^3+^ ions, but may be associated with the features of the framework itself. According to the famous “antenna effect” theory, we know that the emissions are attributed to energy transfer from the ligand to lanthanide ions. If guest molecules enter the framework, energy transfer may be perturbed, thereby, affecting the intensity of the luminescence [39,40,41]. In light of previous research [46,47,48,49], we assume the other possible reason of quenching is that the Fe^3+^ ions and acetone molecules may be adsorbed by the MOFs and may form interactions with the naked Lewis basic pyridyl active sites introduced by the ligand, ultimately resulting in a decrease in the efficiency of the transfer process from the ligand to the lanthanide centers.

In a word, multiple quenching mechanisms may co-work in the Fe^3+^ ions and acetone sensing process. Both competitive energy absorption and the influence of guest molecules on energy transfer may account for the quenching behavior of compounds toward Fe^3+^ ions and acetone.

## 3. Materials and Methods

### 3.1. Chemicals and Reagents

All chemicals and solvents including the H_4_L ligand were purchased commercially and used without further purification.

### 3.2. Apparatus

Powder X-ray diffraction (PXRD) data were recorded using a Rigaku Miniflex 600 diffractometer (Rigaku, Tokyo, Japan). Infrared (IR) spectra using KBr slices were recorded using a PerkinElmer Spectrum One FT-IR (Fourier-transform infrared spectroscopy) spectrometer (PerkinElmer, Dublin, Ireland) ranging from 400 to 4000 cm^−1^. Element analyses (C, H, and N) were measured by a German Elemental Vario EL III instrument (Elementar Analysensysteme GmbH, Langenselbold, Germany). Thermogravimetric analyses (TGA) were measured by a Netzsch STA 449c instrument (Netzsch Corporation, Selb, Germany)and were performed in the temperature range of ambient temperature to 900 °C with a heating rate of 10 °C/min under the flowing nitrogen environment. UV-vis study was recorded on a Lambda 365 (PerkinElmer, Waltham, MA, USA). The photoluminescence spectra of solid-state samples were collected by a Horiba Jobin-Yvon Fluorolog-3 fluorescence spectrometer (HORIBA Jobin Yvon, Kyoto, Japan). An Edinburgh Analytical Instruments FLS920 (Edinburgh Instruments, Edinburgh, UK) was used to capture the fluorescence lifetimes, and the overall photoluminescence quantum yields were investigated by using the integrating sphere covered with barium sulfate at room temperature.

### 3.3. Synthesis of {[Me_2_NH_2_][LnL]·2H_2_O}_n_ (Ln = Eu ***1***, Tb ***2***, Dy ***3***, Gd ***4***) (Compounds ***1***–***4***)

In total, 45 mg of Ln(NO_3_)_3_·6H_2_O and 33 mg of H_4_L were dissolved in DMF (*N*,*N*-dimethylformamide) (5 mL) and H_2_O (5 mL). The solution was sealed in a 25-mL Teflon-lined stainless-steel autoclave, and heated at 160 °C for 72 h. Then the mixture was cooled to room temperature at a rate of 1 °C/min. The resulting colorless crystals, {[Me_2_NH_2_][LnL]·2H_2_O}_n_, were washed with DMF and methanol several times, and then evacuated to remove the co-assembled DMF and methanol in the pores of MOFs. The yields are 68%, 72%, 76%, and 75% for compounds **1**–**4** based on the metal ions. Elemental analysis (%) calculated for the various crystals are listed as follows.

{**[Me_2_NH_2_][EuL]·2H_2_O}_n_ (1)** Elemental analysis (%): calcd for C_16_H_18_EuN_3_O_10_, C, 34.06; H, 3.22; N, 7.45. Found: C, 34.31; H, 3.12; N, 7.50. Infrared (KBr pellet, cm^−1^): 3626(w), 3390(m), 3153(w), 3064(m), 2999(m), 2796(m), 2476(w), 1909(w), 1570(s), 1460(s), 1301(s), 850(s).

{**[Me_2_NH_2_][TbL]·2H_2_O}_n_ (2)** Elemental analysis (%): calcd for C_16_H_18_TbN_3_O_10_, C, 33.64; H, 3.18; N, 7.36. Found: C, 33.74; H, 3.09; N, 7.45. Infrared (KBr pellet, cm^−1^): 3626(w), 3396(m), 3151(w), 3064(m), 2999(m), 2794(m), 2476(w), 1909(w), 1568(s), 1460(s), 1296(s), 850(s).

{**[Me_2_NH_2_][DyL]·2H_2_O}_n_ (3)** Elemental analysis (%): calcd for C_16_H_18_DyN_3_O_10_, C, 33.43; H, 3.16; N, 7.31. Found: C, 33.61; H, 3.04; N, 7.40. Infrared (KBr pellet, cm^−1^): 3622(w), 3394(m), 3151(w), 3064(m), 2999(m), 2794(m), 2474(w), 1911(w), 1568(s), 1460(s), 1301(s), 850(s).

{**[Me_2_NH_2_][GdL]·2H_2_O}_n_ (4)** Elemental analysis (%): calcd for C_16_H_18_GdN_3_O_10_, C, 33.74; H, 3.19; N, 7.38. Found: C, 33.97; H, 3.12; N, 7.46. Infrared (KBr pellet, cm^−1^): 3626(w), 3398(m), 3149(w), 3064(m), 3000(m), 2792(m), 2476(w), 1909(w), 1568(s), 1460(s), 1305(s), 850(s).

### 3.4. Single-Crystal X-ray Crystallography

All the single-crystal X-ray diffraction data for the crystals were recorded on a supernova diffractometer with a multilayer mirror Cu *Kα* radiation (λ = 1.5418 Å) or Mo *K*α (λ = 0.7107 Å). All the structures of compounds **1**–**4** were solved by a direct method of SHELX-97 and full-matrix least-squares refinement on F^2^ was performed using SHELX-97 [50]. Except for some disordered water molecules, all non-hydrogen atoms were anisotropic refined on F^2^ by the full-matrix least-squares technique using the SHELXL-97. Only the [Me_2_NH_2_]^+^ counterion in Gd^3+^ MOF can be solved from single crystal X-ray diffraction. The existences of [Me_2_NH_2_]^+^ in the other three MOFs and water molecules were further verified by the IR spectra, elemental analysis, and TGA data, obtaining the final chemical formulas of compounds **1**–**4**. The CCDC (Cambridge Crystallographic Data Centre) numbers for compounds **1**–**4** are 2043852, 2043854, 2043855, and 2043853, respectively. See Tables S2–S5 for detailed crystallographic data.

### 3.5. Sensing Experiment

In metal ions sensing, 3 mg of activated MOFs was ground to fine powder prepared for the experiments in aqueous solutions. The samples were then dispersed into 3 mL of various M(NO_3_)_x_ aqueous solutions and ultrasonically dispersed to obtain uniform suspensions. Then the suspensions were loaded in the cuvettes for collecting the photoluminescence spectra. The fluorescence intensity of the suspension in water was measured as the blank sample.

In the Tb-MOF paper sensor, uniformly dispersed suspension was obtained via an ultrasonic dispersal of Tb-MOF in di-chloromethane (CH_2_Cl_2_), and then the suspension was evenly spread over the filter paper. After the dichloromethane volatilized and the filter paper dried, the Tb-MOF-coated paper sensor was obtained. The capillary dipped in 0.01 M Fe^3+^ ion aqueous solution was used to write “Fe” on the fluorescent test paper.

In organic solvents sensing, the experiments were carried out by dispersing 3 mg of activated and ground MOFs into 3 mL of different organic solvents. After ultrasonic dispersing the sample evenly, the suspensions were added into the cuvettes and the photoluminescence tests were carried out. The fluorescence intensity of the suspension in DMF was measured as the blank sample.

In acetone vapor sensing, the ground Tb-MOF powder was tiled on the quartz slide to detect acetone vapor. The culture dish containing acetone was put into the inverted crystallizing dish to obtain saturated acetone vapor. After three hours, the quartz slide was put into the inverted crystallizing dish rapidly to make the sample power exposure to the saturated acetone vapor. After the reaction, the quartz slide was put into the fluorescence spectrometer for solid fluorescence detection.

## 4. Conclusions

In summary, a tetradentate carboxylic ligand with naked Lewis basic pyridyl active sites was employed to construct a series of lanthanide MOFs by the solvothermal method. The synthesized compounds have isomorphic structures with the topology point symbol of {4^10^.6^14^.8^4^} {4^5^.6}_2_. Eu-, Tb-, and Dy-MOFs display red, green, and yellow emissions with the maximum peaks at 614, 543, and 574 nm, respectively. The high quantum yields of Eu- and Tb-MOFs suggest that Eu^3+^ and Tb^3+^ can be better sensitized by the ligand than Dy^3+^, which is also proven by their decay lifetimes. The material can detect Fe^3+^ in aqueous solution by a luminescence quenching effect with high selectivity, high sensitivity, and a rapid response (˂40 s). The detection shows excellent anti-inference ability toward many other cations and can be easily recognized by naked eyes. The MOF can also sense acetone solvent and vapor selectively and rapidly. Thus, it may be a rapid luminescent probe for detecting Fe^3+^ and acetone, which can work at complicated circumstances. Furthermore, the framework shows not only good thermal stability but also excellent air and water stabilities, providing a basis for practical applications.

## Figures and Tables

**Table 1:** Crystal data and refinement results for compounds **1***–***4**.

Compound	1	2	3	4
Empirical formula	C_16_H_14_EuN_3_O_8_	C_16_H_14_TbN_3_O_8_	C_16_H_14_DyN_3_O_8_	C_16_H_14_GdN_3_O_8_
Formula weight	528.27	535.23	538.80	533.55
Crystal system	monoclinic	monoclinic	monoclinic	monoclinic
Space group	*P*2_1_/*c*	*P*2_1_/*c*	*P*2_1_/*c*	*P*2_1_/*c*
*a*/Å	9.1791(2)	9.2477(2)	9.1815(5)	9.2303(5)
*b*/Å	10.1636(3)	10.1192(2)	10.1647(4)	10.1530(4)
*c*/Å	21.7390(5)	21.7150(5)	21.7438(9)	21.8214(9)
*α*/°	90	90	90	90
*β*/°	99.829(2)	100.542(2)	99.809(4)	100.353(4)
*γ*/°	90	90	90	90
Volume/Å^3^	1998.32(9)	1997.78(8)	1999.62(16)	2011.70(16)
*Z*	4	4	4	4
*F* (000)	924	909	936	1076
Goodness-of-fit on *F*^2^	1.082	1.021	1.07	1.03
*R*_1_ (I > 2σ (I))	0.0311	0.0282	0.0387	0.0259
*wR*_2_ (reflections)	0.0848	0.0587	0.0701	0.1056

*a, b* and *c* are the unit vectors of unit cell. *α, β*, and *γ* are the unit cell parameters (angles between *a, b* and *c*). Z is the number of molecules in unit cell. F (000) is the total number of electrons in unit cell. *R*_1_ is residual factors. I is diffraction intensity. σ is standard deviation. *w*R_2_ is residual factors.

## Data Availability

Not applicable.

## Supplementary Materials

The following are available online. Figure S1: IR spectra of compounds **1** (a), **2** (b), **3** (c), and **4** (d). Figure S2: The asymmetric unit of **4**. Figure S3: The coordination mode of the [L]^4−^ ligand. Figure S4: PXRD patterns of compounds **1**–**4**. Figure S5: TGA curves of compounds **1**–**4**. Figure S6: (a) X-ray thermodiffractogram, (b) air stability, (c) water stability and (d) pH stability analyses of **2**. Figure S7: (a) The excitation and emission spectra of ligand at room temperature. (b) The emission spectrum of **4** at 77 K. Figure S8: Luminescence decay curves for **1** excited at 297 nm (a) and **2** excited at 299 nm (b) at 298 K. Figure S9: Photoluminescence spectra of **2** in water and aqueous solutions containing different metal ions (1 ×10^−3^ M) when excited at 299 nm. Figure S10: Time-dependent photoluminescence spectra of **2** in Fe^3+^ aqueous solutions (3 × 10^−4^ M). Figure S11: Photoluminescence spectra of **2** in pure water, aqueous solution of Fe^3+^ ion, other cations (Li^+^, Ni^2+^, Mg^2+^, In^3+^, K^+^, Al^3+^, Zn^2+^, Ca^2+^, Cu^2+^) and all the cations, 1 × 10^−3^ M each. Figure S12: Photoluminescence spectra of **2** in different organic solvents when excited at 299 nm. Figure S13: Time-dependent photoluminescence spectra of **2** in the presence of acetone in DMF (0.1 vol.%). Figure S14: PXRD patterns of **2** after dispersed in water and aqueous solutions containing different metal ions. Figure S15: PXRD patterns of **2** after immersed in acetone. Figure S16: UV-vis adsorption spectra of different metal ions in aqueous solution (2 ×10^−4^ M). Figure S17: UV-vis adsorption spectrum of acetone in DMF (0.2 vol.%). Figure S18: UV-vis adsorption spectra of Fe^3+^ aqueous solutions with different concentrations. Figure S19: (a) Photoluminescence spectra of **1** in water and aqueous solutions containing different metal ions (1 ×10^−3^ M) when excited at 297 nm. (b) Comparison of the ^5^D_0_→^7^F_2_ photoluminescence intensity of **1** in water and aqueous solutions con-taining different metal ions when excited at 297 nm. Figure S20: (a) Photoluminescence spectra of **1** in different organic solvents when excited at 297 nm. (b) Comparison of the ^5^D_0_→^7^F_2_ photoluminescence intensity of 1 in different organic solvents when excited at 297 nm. Table S1: Room temperature quantum yields and lifetimes of compounds **1**–**3**. Table S2: Selected bond lengths/Å and bond angles/º for compounds **1**. Table S3: Selected bond lengths/Å and bond angles/º for compounds **2**. Table S4: Selected bond lengths/Å and bond angles/º for compounds **3**. Table S5: Selected bond lengths/Å and bond angles/º for compounds **4**.

## Sample Availability

Samples of the compounds are not available from the authors.
