# Supplementary material for: Construction of a Stable Lanthanide Metal-Organic Framework as a Luminescent Probe for Rapid Naked-Eye Recognition of Fe3+ and Acetone
Source: Molecules. 2021 Mar 18;26(6):1695. doi: 10.3390/molecules26061695 (PMC8003027; doi:10.3390/molecules26061695)

checkCIF/PLATON report

Structure factors have been supplied for datablock(s) 1

THIS REPORT IS FOR GUIDANCE ONLY. IF USED AS PART OF A REVIEW PROCEDURE FOR PUBLICATION, IT SHOULD NOT REPLACE THE EXPERTISE OF AN EXPERIENCED CRYSTALLOGRAPHIC REFEREE.

No syntax errors found. CIF dictionary Interpreting this report

Datablock: 1

```
Bond precision:  C-C = 0.0055 A          Wavelength=1.54184

Cell:            a=9.1791(2)     b=10.1636(3)       c=21.7390(5)
                 alpha=90        beta=99.829(2)     gamma=90
Temperature:     292 K

                Calculated                Reported
Volume          1998.32(9)                1998.32(9)
Space group     P 21/c                    P 1 21/c 1
Hall group      -P 2ybc                   -P 2ybc
Moiety formula  C14 H6 Eu N2 O8           C14 H6 Eu N2 O8
Sum formula     C14 H6 Eu N2 O8           C14 H6 Eu N2 O8
Mr              482.18                    482.17
Dx,g cm-3       1.603                     1.603
Z               4                         4
Mu (mm-1)       22.827                    22.827
F000            924.0                     924.0
F000’           892.11
h,k,lmax        11,12,27                  11,12,26
Nref            4010                      3876
Tmin,Tmax       0.367,0.504               0.161,1.000
Tmin’           0.278

Correction method= # Reported T Limits: Tmin=0.161 Tmax=1.000
AbsCorr = MULTI-SCAN

Data completeness= 0.967          Theta(max)= 73.246

R(reflections)= 0.0311( 3419)     wR2(reflections)= 0.0849( 3876)

S = 1.082                  Npar= 226
```

The following ALERTS were generated. Each ALERT has the format
test-name_ALERT_alert-type_alert-level.
Click on the hyperlinks for more details of the test.

Alert level A

```
PLAT602_ALERT_2_A Solvent Accessible VOID(S) in Structure ........          ! Check
```

Alert level C

```
PLAT911_ALERT_3_C Missing FCF Refl Between Thmin & STh/L=    0.600         25 Report
```

Alert level G

```
PLAT004_ALERT_5_G Polymeric Structure Found with Maximum Dimension          3 Info
PLAT232_ALERT_2_G Hirshfeld Test Diff (M-X)  Eu1      --O5_e     .        5.4 s.u.
PLAT232_ALERT_2_G Hirshfeld Test Diff (M-X)  Eu1      --O7_f     .       18.6 s.u.
PLAT794_ALERT_5_G Tentative Bond Valency for Eu1       (III)     .       3.34 Info
PLAT804_ALERT_5_G Number of ARU-Code Packing Problem(s) in PLATON          11 Info
PLAT883_ALERT_1_G No Info/Value for _atom_sites_solution_primary .     Please Do !
PLAT912_ALERT_4_G Missing # of FCF Reflections Above STh/L=  0.600        109 Note
PLAT933_ALERT_2_G Number of OMIT Records in Embedded .res File ...         12 Note
PLAT941_ALERT_3_G Average HKL Measurement Multiplicity ..........         2.0 Low
PLAT961_ALERT_5_G Dataset Contains no Negative Intensities .......     Please Check
PLAT978_ALERT_2_G Number C-C Bonds with Positive Residual Density.          2 Info
```

---

1 **ALERT level A** = Most likely a serious problem - resolve or explain
0 **ALERT level B** = A potentially serious problem, consider carefully
1 **ALERT level C** = Check. Ensure it is not caused by an omission or oversight
11 **ALERT level G** = General information/check it is not something unexpected

1 ALERT type 1 CIF construction/syntax error, inconsistent or missing data
5 ALERT type 2 Indicator that the structure model may be wrong or deficient
2 ALERT type 3 Indicator that the structure quality may be low
1 ALERT type 4 Improvement, methodology, query or suggestion
4 ALERT type 5 Informative message, check

It is advisable to attempt to resolve as many as possible of the alerts in all categories. Often the minor alerts point to easily fixed oversights, errors and omissions in your CIF or refinement strategy, so attention to these fine details can be worthwhile. In order to resolve some of the more serious problems it may be necessary to carry out additional measurements or structure refinements. However, the purpose of your study may justify the reported deviations and the more serious of these should normally be commented upon in the discussion or experimental section of a paper or in the "special_details" fields of the CIF. checkCIF was carefully designed to identify outliers and unusual parameters, but every test has its limitations and alerts that are not important in a particular case may appear. Conversely, the absence of alerts does not guarantee there are no aspects of the results needing attention. It is up to the individual to critically assess their own results and, if necessary, seek expert advice.

Publication of your CIF in IUCr journals

A basic structural check has been run on your CIF. These basic checks will be run on all CIFs submitted for publication in IUCr journals (*Acta Crystallographica*, *Journal of Applied Crystallography*, *Journal of Synchrotron Radiation*); however, if you intend to submit to *Acta Crystallographica Section C* or *E* or *IUCrData*, you should make sure that full publication checks are run on the final version of your CIF prior to submission.

Publication of your CIF in other journals

Please refer to the *Notes for Authors* of the relevant journal for any special instructions relating to CIF submission.

PLATON version of 05/12/2020; check.def file version of 05/12/2020

Datablock 1 - ellipsoid plot

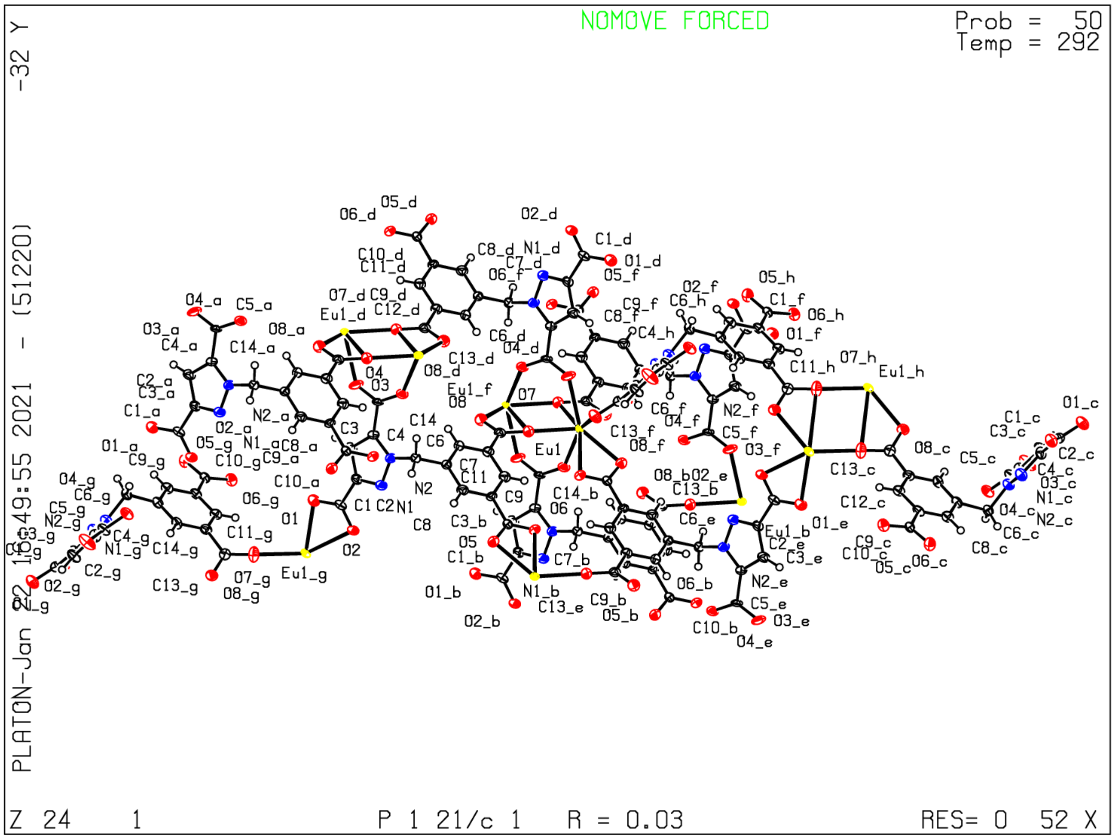

checkCIF/PLATON report

Structure factors have been supplied for datablock(s) 3

THIS REPORT IS FOR GUIDANCE ONLY. IF USED AS PART OF A REVIEW PROCEDURE FOR PUBLICATION, IT SHOULD NOT REPLACE THE EXPERTISE OF AN EXPERIENCED CRYSTALLOGRAPHIC REFEREE.

No syntax errors found. CIF dictionary Interpreting this report

Datablock: 2

```
Bond precision:  C-C = 0.0055 A          Wavelength=1.54184

Cell:            a=9.2477(2)    b=10.1192(2)        c=21.7150(5)
                 alpha=90       beta=100.542(2)     gamma=90
Temperature:     100 K
```

	Calculated	Reported
Volume	1997.78(8)	1997.78(8)
Space group	P 21/c	P 1 21/c 1
Hall group	-P 2ybc	-P 2ybc
Moiety formula	C14 H6 N2 O8 Tb [+ solvent]	C14 H6 N2 O8 Tb
Sum formula	C14 H6 N2 O8 Tb [+ solvent]	C14 H0.25 N2 O8 Tb
Mr	489.14	483.33
Dx,g cm-3	1.626	1.607
Z	4	4
Mu (mm-1)	17.768	17.767
F000	932.0	909.0
F000’	898.84	
h,k,lmax	11,12,26	11,12,26
Nref	4021	3900
Tmin,Tmax	0.396,0.411	0.655,1.000
Tmin’	0.147	

```
Correction method= # Reported T Limits: Tmin=0.655 Tmax=1.000
AbsCorr = MULTI-SCAN

Data completeness= 0.970          Theta(max)= 73.343

R(reflections)= 0.0282( 3212)     wR2(reflections)= 0.0723( 3900)

S = 1.021                  Npar= 226
```

The following ALERTS were generated. Each ALERT has the format
test-name_ALERT_alert-type_alert-level.
Click on the hyperlinks for more details of the test.

Alert level B

```
PLAT430_ALERT_2_B Short Inter D...A Contact  O2       ..O3       .       2.77 Ang.
                                            2-x,1/2+y,1/2-z  =      2_755 Check
```

Alert level C

```
PLAT213_ALERT_2_C Atom O3              has ADP max/min Ratio .....        3.1 prolat
PLAT230_ALERT_2_C Hirshfeld Test Diff for    O3       --C5       .        6.5 s.u.
PLAT250_ALERT_2_C Large U3/U1 Ratio for Average U(i,j) Tensor ....        2.1 Note
PLAT911_ALERT_3_C Missing FCF Refl Between Thmin & STh/L=    0.600          3 Report
PLAT975_ALERT_2_C Check Calcd Resid. Dens.  0.98A   From O7              0.46 eA-3
PLAT975_ALERT_2_C Check Calcd Resid. Dens.  0.88A   From N1              0.43 eA-3
```

Alert level G

```
FORMU01_ALERT_1_G  There is a discrepancy between the atom counts in the
            _chemical_formula_sum and _chemical_formula_moiety. This is
            usually due to the moiety formula being in the wrong format.
            Atom count from _chemical_formula_sum:   C14 H0.25 N2 O8 Tb1
            Atom count from _chemical_formula_moiety:C14 H6 N2 O8 Tb1
FORMU01_ALERT_2_G  There is a discrepancy between the atom counts in the
            _chemical_formula_sum and the formula from the _atom_site* data.
            Atom count from _chemical_formula_sum:C14 H0.25 N2 O8 Tb1
            Atom count from the _atom_site data:  C14 H6 N2 O8 Tb1
CELLZ01_ALERT_1_G Difference between formula and atom_site contents detected.
CELLZ01_ALERT_1_G ALERT: Large difference may be due to a
            symmetry error - see SYMMG tests
           From the CIF: _cell_formula_units_Z    4
           From the CIF: _chemical_formula_sum  C14 H0.25 N2 O8 Tb
           TEST: Compare cell contents of formula and atom_site data

           atom    Z*formula  cif sites diff
           C         56.00     56.00    0.00
           H          1.00     24.00  -23.00
           N          8.00      8.00    0.00
           O         32.00     32.00    0.00
           Tb         4.00      4.00    0.00
PLAT004_ALERT_5_G Polymeric Structure Found with Maximum Dimension          3 Info
PLAT041_ALERT_1_G Calc. and Reported SumFormula    Strings  Differ     Please Check
PLAT068_ALERT_1_G Reported F000 Differs from Calcd (or Missing)...     Please Check
PLAT232_ALERT_2_G Hirshfeld Test Diff (M-X)  Tb1      --O7       .        8.4 s.u.
PLAT232_ALERT_2_G Hirshfeld Test Diff (M-X)  Tb1      --O7_g     .        5.4 s.u.
PLAT432_ALERT_2_G Short Inter X...Y Contact  O3       ..C1               3.00 Ang.
                                           2-x,-1/2+y,1/2-z  =      2_745 Check
PLAT606_ALERT_4_G Solvent Accessible VOID(S) in Structure ........          ! Info
PLAT794_ALERT_5_G Tentative Bond Valency for Tb1       (III)     .       2.97 Info
PLAT912_ALERT_4_G Missing # of FCF Reflections Above STh/L=  0.600        119 Note
PLAT941_ALERT_3_G Average HKL Measurement Multiplicity ..........         1.9 Low
PLAT961_ALERT_5_G Dataset Contains no Negative Intensities .......     Please Check
PLAT978_ALERT_2_G Number C-C Bonds with Positive Residual Density.          1 Info
```

---

0 **ALERT level A** = Most likely a serious problem - resolve or explain
1 **ALERT level B** = A potentially serious problem, consider carefully
6 **ALERT level C** = Check. Ensure it is not caused by an omission or oversight
16 **ALERT level G** = General information/check it is not something unexpected

```
 5 ALERT type 1 CIF construction/syntax error, inconsistent or missing data
11 ALERT type 2 Indicator that the structure model may be wrong or deficient
 2 ALERT type 3 Indicator that the structure quality may be low
 2 ALERT type 4 Improvement, methodology, query or suggestion
 3 ALERT type 5 Informative message, check
```

It is advisable to attempt to resolve as many as possible of the alerts in all categories. Often the minor alerts point to easily fixed oversights, errors and omissions in your CIF or refinement strategy, so attention to these fine details can be worthwhile. In order to resolve some of the more serious problems it may be necessary to carry out additional measurements or structure refinements. However, the purpose of your study may justify the reported deviations and the more serious of these should normally be commented upon in the discussion or experimental section of a paper or in the "special_details" fields of the CIF. checkCIF was carefully designed to identify outliers and unusual parameters, but every test has its limitations and alerts that are not important in a particular case may appear. Conversely, the absence of alerts does not guarantee there are no aspects of the results needing attention. It is up to the individual to critically assess their own results and, if necessary, seek expert advice.

Publication of your CIF in IUCr journals

A basic structural check has been run on your CIF. These basic checks will be run on all CIFs submitted for publication in IUCr journals (*Acta Crystallographica*, *Journal of Applied Crystallography*, *Journal of Synchrotron Radiation*); however, if you intend to submit to *Acta Crystallographica Section C* or *E* or *IUCrData*, you should make sure that full publication checks are run on the final version of your CIF prior to submission.

Publication of your CIF in other journals

Please refer to the *Notes for Authors* of the relevant journal for any special instructions relating to CIF submission.

PLATON version of 05/12/2020; check.def file version of 05/12/2020

Datablock 3 - ellipsoid plot

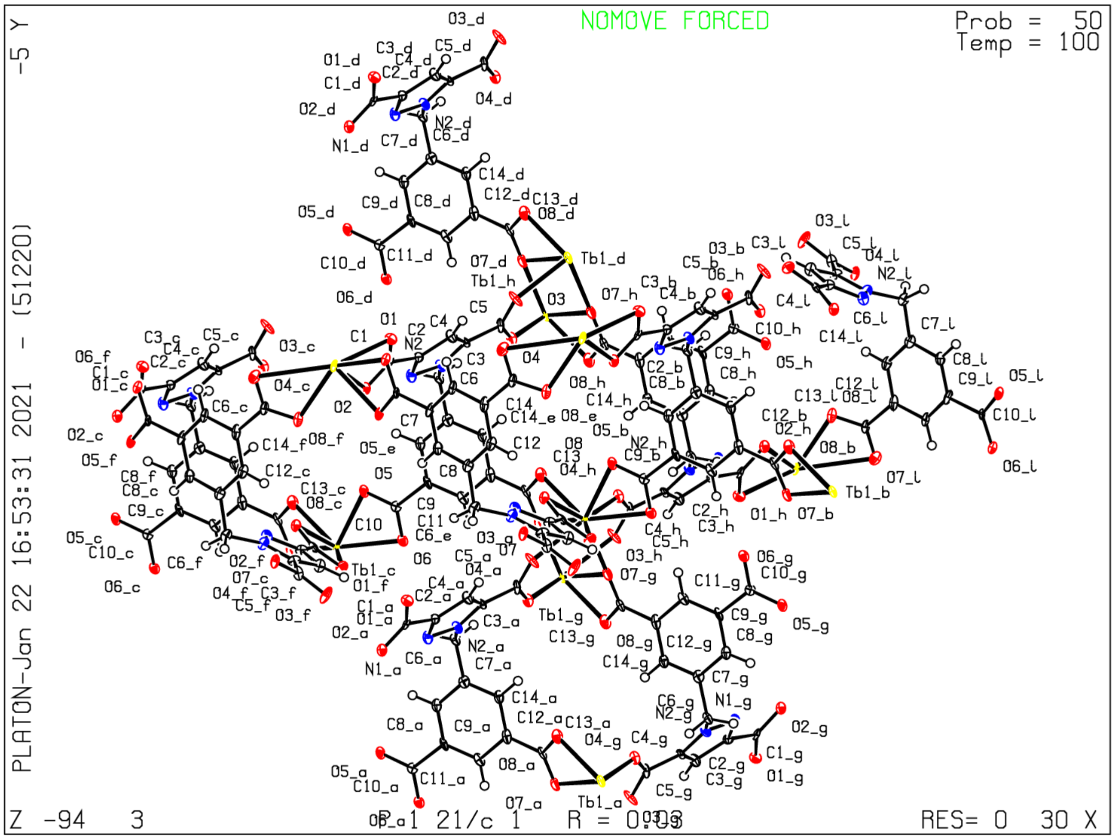

checkCIF/PLATON report

Structure factors have been supplied for datablock(s) 4

THIS REPORT IS FOR GUIDANCE ONLY. IF USED AS PART OF A REVIEW PROCEDURE FOR PUBLICATION, IT SHOULD NOT REPLACE THE EXPERTISE OF AN EXPERIENCED CRYSTALLOGRAPHIC REFEREE.

No syntax errors found. CIF dictionary Interpreting this report

Datablock: 3

```
Bond precision:  C-C = 0.0072 A          Wavelength=1.54184

Cell:            a=9.1815(5)     b=10.1647(4)       c=21.7438(9)
                 alpha=90        beta=99.809(4)     gamma=90
Temperature:     293 K

                Calculated                Reported
Volume          1999.62(16)               1999.62(16)
Space group     P 21/c                    P 1 21/c 1
Hall group      -P 2ybc                   -P 2ybc
Moiety formula  C14 H6 Dy N2 O8           C14 H6 Dy N2 O8
Sum formula     C14 H6 Dy N2 O8           C14 H6 Dy N2 O8
Mr              492.71                    492.71
Dx,g cm-3       1.637                     1.637
Z               4                         4
Mu (mm-1)       20.352                    20.352
F000            936.0                     936.0
F000'           900.32
h,k,lmax        11,12,27                  11,12,26
Nref            4010                      3879
Tmin,Tmax       0.130,0.131               0.278,1.000
Tmin'           0.011

Correction method= # Reported T Limits: Tmin=0.278 Tmax=1.000
AbsCorr = MULTI-SCAN

Data completeness= 0.967          Theta(max)= 73.247

R(reflections)= 0.0387( 3133)     wR2(reflections)= 0.1149( 3879)

S = 1.070                  Npar= 226
```

The following ALERTS were generated. Each ALERT has the format
test-name_ALERT_alert-type_alert-level.
Click on the hyperlinks for more details of the test.

Alert level A

```
PLAT602_ALERT_2_A Solvent Accessible VOID(S) in Structure ........          ! Check
```

Alert level C

```
PLAT911_ALERT_3_C Missing FCF Refl Between Thmin & STh/L=    0.600         21 Report
PLAT918_ALERT_3_C Reflection(s) with I(obs) much Smaller I(calc) .          1 Check
PLAT934_ALERT_3_C Number of (Iobs-Icalc)/Sigma(W) > 10 Outliers ..          1 Check
PLAT972_ALERT_2_C Check Calcd Resid. Dens.  0.89A   From Dy1            -1.67 eA-3
PLAT972_ALERT_2_C Check Calcd Resid. Dens.  0.97A   From Dy1            -1.56 eA-3
PLAT975_ALERT_2_C Check Calcd Resid. Dens.  0.85A   From O7              0.78 eA-3
PLAT976_ALERT_2_C Check Calcd Resid. Dens.  0.86A   From O2             -0.52 eA-3
```

Alert level G

```
PLAT003_ALERT_2_G Number of Uiso or Uij Restrained non-H Atoms ...          3 Report
PLAT004_ALERT_5_G Polymeric Structure Found with Maximum Dimension          3 Info
PLAT177_ALERT_4_G The CIF-Embedded .res File Contains DELU Records          2 Report
PLAT178_ALERT_4_G The CIF-Embedded .res File Contains SIMU Records          2 Report
PLAT186_ALERT_4_G The CIF-Embedded .res File Contains ISOR Records          1 Report
PLAT199_ALERT_1_G Reported _cell_measurement_temperature ..... (K)        293 Check
PLAT200_ALERT_1_G Reported   _diffrn_ambient_temperature ..... (K)        293 Check
PLAT794_ALERT_5_G Tentative Bond Valency for Dy1       (II)      .       2.10 Info
PLAT804_ALERT_5_G Number of ARU-Code Packing Problem(s) in PLATON          11 Info
PLAT860_ALERT_3_G Number of Least-Squares Restraints .............         20 Note
PLAT912_ALERT_4_G Missing # of FCF Reflections Above STh/L=  0.600        111 Note
PLAT933_ALERT_2_G Number of OMIT Records in Embedded .res File ...         11 Note
PLAT941_ALERT_3_G Average HKL Measurement Multiplicity ...........        2.0 Low
PLAT961_ALERT_5_G Dataset Contains no Negative Intensities .......     Please Check
PLAT978_ALERT_2_G Number C-C Bonds with Positive Residual Density.          0 Info
```

```
   1 ALERT level A = Most likely a serious problem - resolve or explain
   0 ALERT level B = A potentially serious problem, consider carefully
   7 ALERT level C = Check. Ensure it is not caused by an omission or oversight
  15 ALERT level G = General information/check it is not something unexpected

   2 ALERT type 1 CIF construction/syntax error, inconsistent or missing data
   8 ALERT type 2 Indicator that the structure model may be wrong or deficient
   5 ALERT type 3 Indicator that the structure quality may be low
   4 ALERT type 4 Improvement, methodology, query or suggestion
   4 ALERT type 5 Informative message, check
```

It is advisable to attempt to resolve as many as possible of the alerts in all categories. Often the minor alerts point to easily fixed oversights, errors and omissions in your CIF or refinement strategy, so attention to these fine details can be worthwhile. In order to resolve some of the more serious problems it may be necessary to carry out additional measurements or structure refinements. However, the purpose of your study may justify the reported deviations and the more serious of these should normally be commented upon in the discussion or experimental section of a paper or in the "special_details" fields of the CIF. checkCIF was carefully designed to identify outliers and unusual parameters, but every test has its limitations and alerts that are not important in a particular case may appear. Conversely, the absence of alerts does not guarantee there are no aspects of the results needing attention. It is up to the individual to critically assess their own results and, if necessary, seek expert advice.

Publication of your CIF in IUCr journals

A basic structural check has been run on your CIF. These basic checks will be run on all CIFs submitted for publication in IUCr journals (*Acta Crystallographica*, *Journal of Applied Crystallography*, *Journal of Synchrotron Radiation*); however, if you intend to submit to *Acta Crystallographica Section C* or *E* or *IUCrData*, you should make sure that full publication checks are run on the final version of your CIF prior to submission.

Publication of your CIF in other journals

Please refer to the *Notes for Authors* of the relevant journal for any special instructions relating to CIF submission.

PLATON version of 05/12/2020; check.def file version of 05/12/2020

Datablock 4 - ellipsoid plot

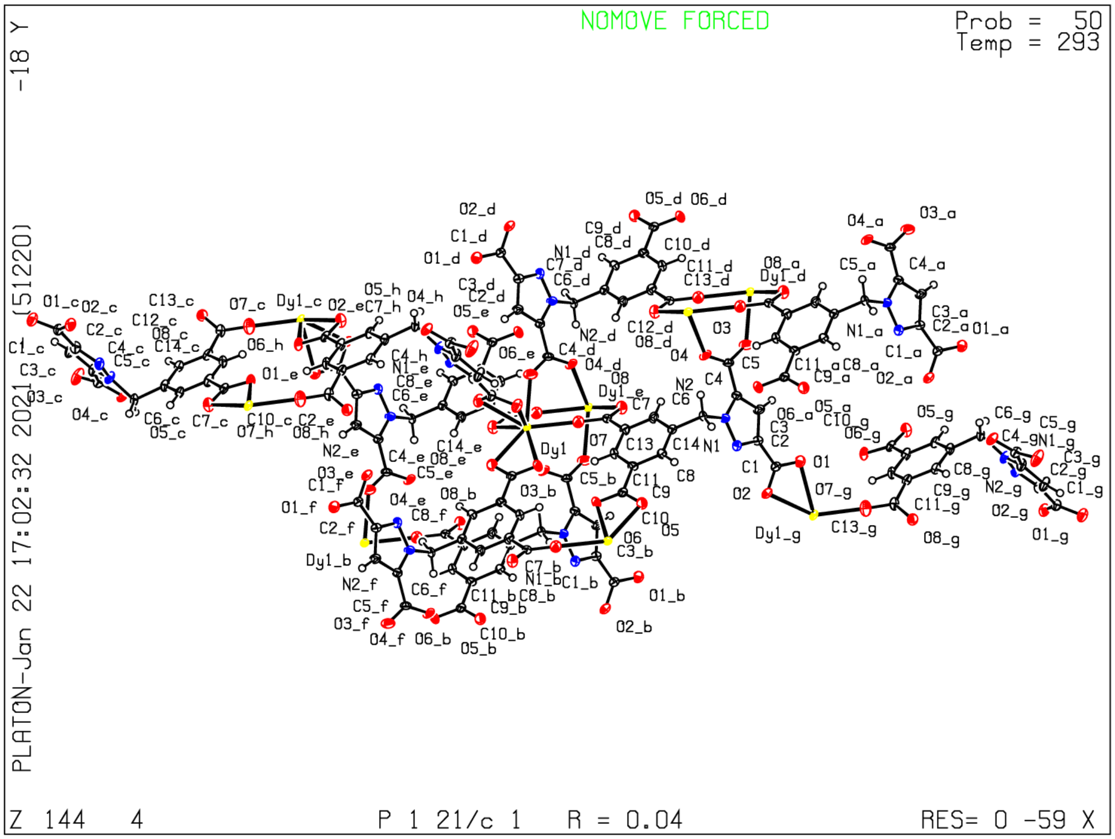

checkCIF/PLATON report

Structure factors have been supplied for datablock(s) 2

THIS REPORT IS FOR GUIDANCE ONLY. IF USED AS PART OF A REVIEW PROCEDURE FOR PUBLICATION, IT SHOULD NOT REPLACE THE EXPERTISE OF AN EXPERIENCED CRYSTALLOGRAPHIC REFEREE.

No syntax errors found. CIF dictionary Interpreting this report

Datablock: 4

```
Bond precision:  C-C = 0.0050 A          Wavelength=0.71073

Cell:            a=9.2303(5)    b=10.1530(4)        c=21.8214(9)
                 alpha=90       beta=100.353(4)     gamma=90
Temperature:     134 K

                Calculated                Reported
Volume          2011.70(16)               2011.70(16)
Space group     P 21/c                    P 1 21/c 1
Hall group      -P 2ybc                   -P 2ybc
Moiety formula  C14 H6 Gd N2 O8, C2 H8 N  C14 H6 Gd N2 O8, C2 H8 N
Sum formula     C16 H14 Gd N3 O8          C16 H14 Gd N3 O8
Mr              533.55                    533.55
Dx,g cm-3       1.762                     1.762
Z               4                         4
Mu (mm-1)       3.343                     3.343
F000            1036.0                    1036.0
F000'           1035.82
h,k,lmax        11,12,27                  11,12,27
Nref            4112                      4091
Tmin,Tmax       0.676,0.716               0.773,1.000
Tmin'           0.507

Correction method= # Reported T Limits: Tmin=0.773 Tmax=1.000
AbsCorr = MULTI-SCAN

Data completeness= 0.995          Theta(max)= 26.372

R(reflections)= 0.0259( 3385)     wR2(reflections)= 0.0573( 4091)

S = 1.039                    Npar= 255
```

The following ALERTS were generated. Each ALERT has the format
test-name_ALERT_alert-type_alert-level.
Click on the hyperlinks for more details of the test.

Alert level C

```
PLAT244_ALERT_4_C Low    'Solvent' Ueq as Compared to Neighbors of         N3 Check
PLAT420_ALERT_2_C D-H Without Acceptor       N3       --H3B      .     Please Check
PLAT601_ALERT_2_C Unit Cell Contains Solvent Accessible VOIDS of .         60 Ang**3
PLAT910_ALERT_3_C Missing # of FCF Reflection(s) Below Theta(Min).         10 Note
PLAT911_ALERT_3_C Missing FCF Refl Between Thmin & STh/L=    0.600         12 Report
PLAT975_ALERT_2_C Check Calcd Resid. Dens.  0.93A   From N1              0.51 eA-3
```

Alert level G

```
PLAT004_ALERT_5_G Polymeric Structure Found with Maximum Dimension          3 Info
PLAT007_ALERT_5_G Number of Unrefined Donor-H Atoms .............           2 Report
PLAT232_ALERT_2_G Hirshfeld Test Diff (M-X)  Gd1      --O7_e     .        7.2 s.u.
PLAT794_ALERT_5_G Tentative Bond Valency for Gd1       (III)     .       3.14 Info
PLAT804_ALERT_5_G Number of ARU-Code Packing Problem(s) in PLATON          11 Info
PLAT933_ALERT_2_G Number of OMIT Records in Embedded .res File ...         13 Note
PLAT941_ALERT_3_G Average HKL Measurement Multiplicity ...........        4.1 Low
PLAT978_ALERT_2_G Number C-C Bonds with Positive Residual Density.          1 Info
```

---

0 **ALERT level A** = Most likely a serious problem - resolve or explain
0 **ALERT level B** = A potentially serious problem, consider carefully
6 **ALERT level C** = Check. Ensure it is not caused by an omission or oversight
8 **ALERT level G** = General information/check it is not something unexpected

0 ALERT type 1 CIF construction/syntax error, inconsistent or missing data
6 ALERT type 2 Indicator that the structure model may be wrong or deficient
3 ALERT type 3 Indicator that the structure quality may be low
1 ALERT type 4 Improvement, methodology, query or suggestion
4 ALERT type 5 Informative message, check

It is advisable to attempt to resolve as many as possible of the alerts in all categories. Often the minor alerts point to easily fixed oversights, errors and omissions in your CIF or refinement strategy, so attention to these fine details can be worthwhile. In order to resolve some of the more serious problems it may be necessary to carry out additional measurements or structure refinements. However, the purpose of your study may justify the reported deviations and the more serious of these should normally be commented upon in the discussion or experimental section of a paper or in the "special_details" fields of the CIF. checkCIF was carefully designed to identify outliers and unusual parameters, but every test has its limitations and alerts that are not important in a particular case may appear. Conversely, the absence of alerts does not guarantee there are no aspects of the results needing attention. It is up to the individual to critically assess their own results and, if necessary, seek expert advice.

Publication of your CIF in IUCr journals

A basic structural check has been run on your CIF. These basic checks will be run on all CIFs submitted for publication in IUCr journals (*Acta Crystallographica*, *Journal of Applied Crystallography*, *Journal of Synchrotron Radiation*); however, if you intend to submit to *Acta Crystallographica Section C* or *E* or *IUCrData*, you should make sure that full publication checks are run on the final version of your CIF prior to submission.

Publication of your CIF in other journals

Please refer to the *Notes for Authors* of the relevant journal for any special instructions relating to CIF submission.

PLATON version of 05/12/2020; check.def file version of 05/12/2020

Datablock 2 - ellipsoid plot

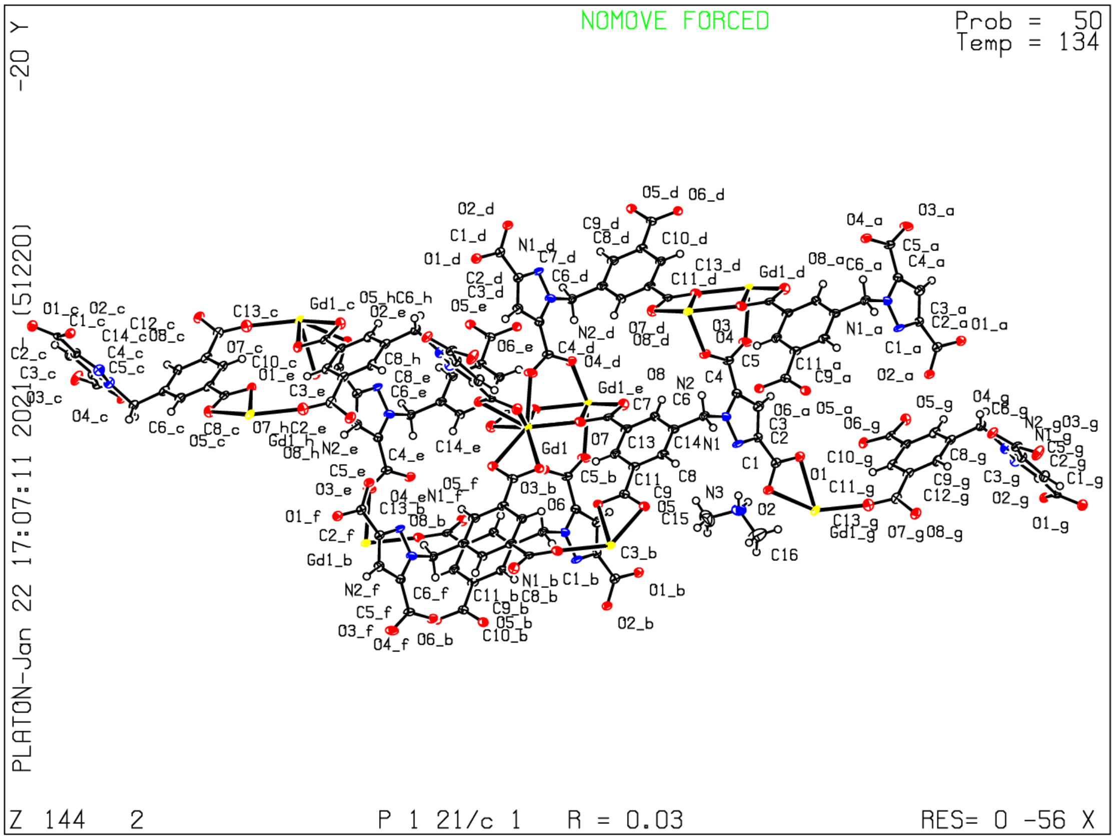

Supplement: Supplementary file 1 [file molecules-26-01695-s001.zip › molecules-1124799-supplementary/manuscript-supplementary-revised/checkcif.pdf]
