# Supplementary material for: Construction of a Stable Lanthanide Metal-Organic Framework as a Luminescent Probe for Rapid Naked-Eye Recognition of Fe3+ and Acetone
Source: Molecules. 2021 Mar 18;26(6):1695. doi: 10.3390/molecules26061695 (PMC8003027; doi:10.3390/molecules26061695)
Supplement: Supplementary file 1 [file molecules-26-01695-s001.zip › molecules-1124799-supplementary/manuscript-supplementary-revised/explanation for check cif.docx]

PLAT430_ALERT_2_B Short Inter D...A Contact O9 ..O21 . 2.77 Ang.

2-x,1/2+y,1/2-z = 2_755 Check

These close contacts arise between deprotonated carboxylic acids from neighbor ligands. The oxygen atoms are all coordinated with the Tb^3+^ ions, so we think there are probably no hydrogen bonds between O9 and O21.

PLAT602_ALERT_2_A Solvent Accessible VOID(S) in Structure

In the structures, large volume fraction is occupied with solvent molecules. Some of solvent molecules are severely disordered and thus not included into refinement.
