# Supplementary material for: Construction of a Stable Lanthanide Metal-Organic Framework as a Luminescent Probe for Rapid Naked-Eye Recognition of Fe3+ and Acetone
Source: Molecules. 2021 Mar 18;26(6):1695. doi: 10.3390/molecules26061695 (PMC8003027; doi:10.3390/molecules26061695)
Supplement: Supplementary file 1 [file molecules-26-01695-s001.zip › molecules-1124799-supplementary/manuscript-supplementary-revised/molecules-1124799-revised-supplementary.doc]

Supplementary Material

Construction of a Stable Lanthanide Metal-Organic Framework as a Luminescent Probe for Rapid Naked-Eye Recognition of Fe^3+^ and Acetone

Jiayishuo Wang ^1,2^, Muxin Yu ^1,3^, Lian Chen ^1^*, Zhijia Li ^1,2^, Shengchang Li ^1,2^, Feilong Jiang ^1^ and Maochun Hong ^1^*

^1^	Key Laboratory of Optoelectronic Materials Chemistry and Physics, Fujian Institute of Research on the Structure of Matter, Chinese Academy of Sciences, Fuzhou, 350002, China; wangjiayishuo@fjirsm.ac.cn (J.W.); muxinyu@fjirsm.ac.cn (M.Y.); lizhijia@fjirsm.ac.cn (Z.L.); lishengchang@fjirsm.ac.cn (S.L.); fjiang@fjirsm.ac.cn (F.J.)

^2^	University of the Chinese Academy of Sciences, Beijing, 100049, China

^3^	Organic Optoelectronics Engineering Research Centre of Fujian’s Universities, College of Electronics and Information Science, Fujian Jiangxia University, Fuzhou, 350108, China

*	Correspondence: cl@fjirsm.ac.cn (L.C.); hmc@fjirsm.ac.cn (M.H.)

Contents

Figure S1. IR spectra of compounds **1** (a), **2** (b), **3** (c), and **4** (d). ..………………… 3

Figure S2. The asymmetric unit of **4**. …………………….…............................……..3

Figure S3. The coordination mode of the [L]^4-^ ligand. …………………………….…4

Figure S4. PXRD patterns of compounds **1**-**4**. ……………………………….….…...4

Figure S5. TGA curves of compounds **1**-**4**. ………………………………...…..…….5

Figure S6. (a) X-ray thermodiffractogram, (b) air stability, (c) water stability and (d) pH stability analyses of **2**. ………………………………………………………...….5

Figure S7. (a) The excitation and emission spectra of ligand at room temperature. (b) The emission spectrum of **4** at 77 K. ……………………………………………….....6

Figure S8. Luminescence decay curves for 1 excited at 297 nm (a) and **2** excited at 299 nm (b) at 298 K. …………………..………………………................………..….6

Figure S9. Photoluminescence spectra of **2** in water and aqueous solutions containing different metal ions (1 ×10^-3^ M) when excited at 299 nm. ………………………...…6

Figure S10. Time-dependent photoluminescence spectra of **2** in Fe^3+^ aqueous solutions (3 × 10^-4^ M). .………......………………………………………………..…..7

Figure S11. Photoluminescence spectra of **2** in pure water, aqueous solution of Fe^3+^ ion, other cations (Li^+^, Ni^2+^, Mg^2+^, In^3+^, K^+^, Al^3+^, Zn^2+^, Ca^2+^, Cu^2+^) and all the cations, 1 × 10^-3^ M each. …………………….……………….……………….…………….…7

Figure S12. Photoluminescence spectra of **2** in different organic solvents when excited at 299 nm. …………………………………………………...……………..…8

Figure S13. Time-dependent photoluminescence spectra of **2** in the presence of acetone in DMF (0.1 vol%). …………….............………………………………........8

Figure S14. PXRD patterns of **2** after dispersed in water and aqueous solutions containing different metal ions. ……………………..…………………….……...….9

Figure S15. PXRD patterns of **2** after immersed in acetone. ………………….……9

Figure S16. UV-vis adsorption spectra of different metal ions in aqueous solution (2 ×10^-4^ M). ......................................................................................................................10

Figure S17. UV-vis adsorption spectrum of acetone in DMF (0.2 vol%). …..….......10

Figure S18. UV-vis adsorption spectra of Fe^3+^ aqueous solutions with different concentrations. ……………………………………………………………………….11

Figure S19. (a) Photoluminescence spectra of **1** in water and aqueous solutions containing different metal ions (1 ×10^−3^ M) when excited at 297 nm. (b) Comparison of the ^5^D_0_→^7^F_2_ photoluminescence intensity of **1** in water and aqueous solutions containing different metal ions when excited at 297 nm. .......11

Figure S20. (a) Photoluminescence spectra of **1** in different organic solvents when excited at 297 nm. (b) Comparison of the ^5^D_0_→^7^F_2_ photoluminescence intensity of **1** in different organic solvents when excited at 297 nm. ……………………………...….12

Table S1. Room temperature quantum yields and lifetimes of compounds **1**-**3**…......12

Table S2. Selected bond lengths/Å and bond angles/º for compounds **1**. …….…..12

Table S3. Selected bond lengths/Å and bond angles/º for compounds **2**. …….…..12

Table S4. Selected bond lengths/Å and bond angles/º for compounds **3**. …….…..13

Table S5. Selected bond lengths/Å and bond angles/º for compounds **4**. …….…..13

**Figure S1.** IR spectra of compounds **1** (**a**), **2** (**b**), **3** (**c**), and **4** (**d**).

**Figure S2.** The asymmetric unit of **4**. (Gd, green; O, red; N, blue; C, gray).

**Figure S3.** The coordination mode of the [L]^4−^ ligand.

**Figure S4.** PXRD patterns of compounds **1–4**.

**Figure S5.** TGA curves of compounds **1–4**.

**Figure S6.** (**a**) X-ray thermodiffractogram, (**b**) air stability, (**c**) water stability and (**d**) pH stability analyses of **2**.

**Figure S7.** (**a**) The excitation and emission spectra of ligand at room temperature. (**b**) The emission spectrum of **4** at 77 K.

**Figure S8.** Luminescence decay curves for **1** excited at 297 nm (**a**) and **2** excited at 299 nm (**b**) at 298 K.

**Figure S9.** Photoluminescence spectra of **2** in water and aqueous solutions containing different metal ions (1 ×10^−3^ M) when excited at 299 nm.

**Figure S10.** Time-dependent photoluminescence spectra of **2** in Fe^3+^ aqueous solutions (3 × 10^−4^ M).

**Figure S11.** Photoluminescence spectra of **2** in pure water, aqueous solution of Fe^3+^ ion, other cations (Li^+^, Ni^2+^, Mg^2+^, In^3+^, K^+^, Al^3+^, Zn^2+^, Ca^2+^, Cu^2+^) and all the cations, 1 × 10^−3^ M each.

**Figure S12.** Photoluminescence spectra of **2** in different organic solvents when excited at 299 nm.

**Figure S13.** Time-dependent photoluminescence spectra of **2** in the presence of acetone in DMF (0.1 vol%).

**Figure S14.** PXRD patterns of **2** after dispersed in water and aqueous solutions containing different metal ions.

**Figure S15.** PXRD patterns of **2** after immersed in acetone.

**Figure S16.** UV-vis adsorption spectra of different metal ions in aqueous solution (2 ×10^−4^ M).

**Figure S17.** UV-vis adsorption spectrum of acetone in DMF (0.2 vol%).

**Figure S18.** UV-vis adsorption spectra of Fe^3+^ aqueous solutions with different concentrations.

**Figure S19.** (**a**) Photoluminescence spectra of **1** in water and aqueous solutions containing different metal ions (1 ×10^−3^ M) when excited at 297 nm. (**b**) Comparison of the ^5^D_0_→^7^F_2_ photoluminescence intensity of **1** in water and aqueous solutions containing different metal ions when excited at 297 nm.

**Figure S20.** (**a**) Photoluminescence spectra of **1** in different organic solvents when excited at 297 nm. (**b**) Comparison of the ^5^D_0_→^7^F_2_ photoluminescence intensity of **1** in different organic solvents when excited at 297 nm.

**Table S1.** Room temperature quantum yields and lifetimes of compounds **1-3**.

| **Compound** | **1** | **2** | **3** |
| --- | --- | --- | --- |
| *Φ*_overall_ (%) | 39.29 | 57.77 | <1 |
| τ_obs_ (ms) | 0.9785 | 1.093 | 1.095 × 10^−2^ |

**Table S2.** Selected bond lengths/Å and bond angles/º for compounds **1**.

| **Bond** | **Dist.** | **Bond** | **Dist.** |
| --- | --- | --- | --- |
| Eu1—O4^1^ | 2.383(3) | Eu1—O5^2^ | 2.376(3) |
| Eu1—O6^2^ | 2.444(3) | Eu1—O3^5^ | 2.366(3) |
| Eu1—O1^3^ | 2.446(3) | Eu1—O7^4^ | 2.929(3) |
| Eu1—O8^4^ | 2.380(3) | Eu1—O7 | 2.295(3) |
| Eu1—O2^3^ | 2.530(3) |  |  |
| **Angle** | **(º)** | **Angle** | **(º)** |
| O4^1^—Eu1—O6^2^ | 75.40(10) | O5^2^—Eu1—O8^4^ | 83.21(10) |
| O4^1^—Eu1—O1^3^ | 146.34(9) | O5^2^—Eu1—O2^3^ | 72.25(9) |
| O4^1^—Eu1—O2^3^ | 142.93(10) | O5^2^—Eu1—O7^4^ | 124.82(9) |
| O4^1^—Eu1—O7^4^ | 68.21(9) | O3^5^—Eu1—O4^1^ | 133.28(9) |
| O6^2^—Eu1—O1^3^ | 70.97(10) | O3^5^—Eu1—O6^2^ | 140.11(11) |
| O6^2^—Eu1—O2^3^ | 105.37(9) | O3^5^—Eu1—O1^3^ | 76.19(10) |
| O6^2^—Eu1—O7^4^ | 142.35(9) | O3^5^—Eu1—O8^4^ | 85.64(11) |
| O1^3^—Eu1—O2^3^ | 52.07(9) | O3^5^—Eu1—O2^3^ | 69.30(9) |
| O1^3^—Eu1—O7^4^ | 144.32(9) | O3^5^—Eu1—O5^2^ | 141.51(9) |
| O8^4^—Eu1—O4^1^ | 77.57(10) | O3^5^—Eu1—O7^4^ | 68.31(10) |
| O8^4^—Eu1—O6^2^ | 132.69(9) | O7—Eu1—O4^1^ | 76.52(11) |
| O8^4^—Eu1—O1^3^ | 127.69(9) | O7—Eu1—O6^2^ | 82.45(9) |
| O8^4^—Eu1—O2^3^ | 75.63(9) | O7—Eu1—O1^3^ | 97.57(11) |
| O8^4^—Eu1—O7^4^ | 47.61(8) | O7—Eu1—O8^4^ | 127.24(10) |
| O2^3^—Eu1—O7^4^ | 109.33(8) | O7—Eu1—O2^3^ | 140.53(10) |
| O5^2^—Eu1—O4^1^ | 79.59(9) | O7—Eu1—O5^2^ | 134.60(10) |
| O5^2^—Eu1—O6^2^ | 54.25(9) | O7—Eu1—O3^5^ | 80.05(10) |
| O5^2^—Eu1—O1^3^ | 81.92(10) | O7—Eu1—O7^4^ | 80.11(10) |

Symmetry transformations used to generate equivalent atoms: ^1^1+x,+y,+z; ^2^2-x,1-y,1-z; ^3^1+x,3/2-y,1/2+z; ^4^2-x,2-y,1-z; ^5^1-x,2-y,1-z.

**Table S3.** Selected bond lengths/Å and bond angles/º for compounds **2**.

| **Bond** | **Dist.** | **Bond** | **Dist.** |
| --- | --- | --- | --- |
| Tb1—O7^1^ | 2.302(3) | Tb1—O8 | 2.378(3) |
| Tb1—O7 | 2.829(3) | Tb1—O2^4^ | 2.515(2) |
| Tb1—O5^2^ | 2.379(3) | Tb1—O3^5^ | 2.361(2) |
| Tb1—O4^3^ | 2.386(2) | Tb1—O1^4^ | 2.457(2) |
| Tb1—O6^2^ | 2.456(3) |  |  |
| **Angle** | **(º)** | **Angle** | **(º)** |
| O7^1^—Tb1—O7 | 79.11(10) | O6^2^—Tb1—O2^4^ | 105.84(8) |
| O7^1^—Tb1—O5^2^ | 135.27(9) | O6^2^—Tb1—O1^4^ | 70.46(8) |
| O7^1^—Tb1—O4^3^ | 76.55(9) | O8—Tb1—O7 | 49.01(8) |
| O7^1^—Tb1—O6^2^ | 83.04(9) | O8—Tb1—O5^2^ | 82.20(9) |
| O7^1^—Tb1—O8 | 127.54(9) | O8—Tb1—O4^3^ | 77.77(9) |
| O7^1^—Tb1—O2^4^ | 141.27(8) | O8—Tb1—O6^2^ | 131.56(9) |
| O7^1^—Tb1—O3^5^ | 79.80(9) | O8—Tb1—O2^4^ | 74.51(8) |
| O7^1^—Tb1—O1^4^ | 98.49(9) | O8—Tb1—O1^4^ | 127.30(8) |
| O5^2^—Tb1—O7 | 125.47(8) | O2^4^—Tb1—O7 | 108.97(8) |
| O5^2^—Tb1—O4^3^ | 79.49(9) | O3^5^—Tb1—O7 | 69.07(8) |
| O5^2^—Tb1—O6^2^ | 54.25(8) | O3^5^—Tb1—O5^2^ | 140.72(9) |
| O5^2^—Tb1—O2^4^ | 71.68(8) | O3^5^—Tb1—O4^3^ | 134.77(9) |
| O5^2^—Tb1—O1^4^ | 80.67(9) | O3^5^—Tb1—O6^2^ | 139.41(10) |
| O4^3^—Tb1—O7 | 68.95(8) | O3^5^—Tb1—O8 | 87.22(10) |
| O4^3^—Tb1—O6^2^ | 74.62(8) | O3^5^—Tb1—O2^4^ | 69.04(8) |
| O4^3^—Tb1—O2^4^ | 142.13(8) | O3^5^—Tb1—O1^4^ | 76.08(9) |
| O4^3^—Tb1—O1^4^ | 145.07(9) | O1^4^—Tb1—O7 | 144.97(8) |
| O6^2^—Tb1—O7 | 142.19(8) | O1^4^—Tb1—O2^4^ | 52.80(8) |

Symmetry transformations used to generate equivalent atoms: ^1^-x,1-y,-z; ^2^+x,-1+y,+z; ^3^1-x,1-y,-z; ^4^1-x,-1/2+y,1/2-z; ^5^-1+x,+y,+z.

**Table S4.** Selected bond lengths/Å and bond angles/º for compounds **3**.

| **Bond** | **Dist.** | **Bond** | **Dist.** |
| --- | --- | --- | --- |
| Dy1—O6^1^ | 2.442(3) | Dy1—O2^4^ | 2.530(4) |
| Dy1—O4^2^ | 2.388(3) | Dy1—O1^4^ | 2.444(4) |
| Dy1—O8^3^ | 2.384(4) | Dy1—O3^5^ | 2.359(4) |
| Dy1—O5^1^ | 2.366(4) | Dy1—O7 | 2.293(4) |
| **Angle** | **(º)** | **Angle** | **(º)** |
| O6^1^—Dy1—O2^2^ | 105.38(13) | O1^2^—Dy1—O2^2^ | 52.31(12) |
| O6^1^—Dy1—O1^2^ | 70.91(13) | O3^5^—Dy1—O6^1^ | 139.82(15) |
| O4^3^—Dy1—O6^1^ | 75.37(13) | O3^5^—Dy1—O4^3^ | 133.32(13) |
| O4^3^—Dy1—O2^2^ | 142.90(14) | O3^5^—Dy1—O8^4^ | 85.97(15) |
| O4^3^—Dy1—O1^2^ | 146.24(12) | O3^5^—Dy1—O5^1^ | 141.69(14) |
| O8^4^—Dy1—O6^1^ | 132.64(13) | O3^5^—Dy1—O2^2^ | 69.50(13) |
| O8^4^—Dy1—O4^3^ | 77.70(13) | O3^5^—Dy1—O1^2^ | 76.03(14) |
| O8^4^—Dy1—O2^2^ | 75.45(12) | O7—Dy1—O6^1^ | 82.67(13) |
| O8^4^—Dy1—O1^2^ | 127.75(13) | O7—Dy1—O4^3^ | 76.62(15) |
| O5^1^—Dy1—O6^1^ | 54.48(13) | O7—Dy1—O8^4^ | 127.27(14) |
| O5^1^—Dy1—O4^3^ | 79.46(13) | O7—Dy1—O5^1^ | 134.99(13) |
| O5^1^—Dy1—O8^4^ | 82.83(13) | O7—Dy1—O2^2^ | 140.45(14) |
| O5^1^—Dy1—O2^2^ | 72.23(13) | O7—Dy1—O1^2^ | 97.37(15) |
| O5^1^—Dy1—O1^2^ | 82.29(14) | O7—Dy1—O3^5^ | 79.55(14) |

Symmetry transformations used to generate equivalent atoms: ^1^2-x,1-y,1-z; ^2^1+x,+y,+z; ^3^2-x,-y,1-z; ^4^1+x,1/2-y,1/2+z; ^5^1-x,-y,1-z.

**Table S5.** Selected bond lengths/Å and bond angles/º for compounds **4**.

| **Bond** | **Dist.** | **Bond** | **Dist.** |
| --- | --- | --- | --- |
| Gd1—O2^1^ | 2.545(2) | Gd1—O5^4^ | 2.396(2) |
| Gd1—O4^2^ | 2.412(2) | Gd1—O1^1^ | 2.476(2) |
| Gd1—O7^3^ | 2.814(2) | Gd1—O3^5^ | 2.390(2) |
| Gd1—O7 | 2.342(3) | Gd1—O8^3^ | 2.418(2) |
| Gd1—O6^4^ | 2.475(2) |  |  |
| **Angle** | **(º)** | **Angle** | **(º)** |
| O2^1^—Gd1—O7^2^ | 108.06(7) | O5^4^—Gd1—O4^3^ | 79.60(8) |
| O4^3^—Gd1—O2^1^ | 142.21(8) | O5^4^—Gd1—O7^2^ | 125.62(7) |
| O4^3^—Gd1—O7^2^ | 69.60(7) | O5^4^—Gd1—O6^4^ | 53.73(8) |
| O4^3^—Gd1—O6^4^ | 74.58(8) | O5^4^—Gd1—O1^1^ | 79.70(8) |
| O4^3^—Gd1—O1^1^ | 145.18(8) | O5^4^—Gd1—O8^2^ | 81.64(8) |
| O4^3^—Gd1—O8^2^ | 77.49(8) | O1^1^—Gd1—O2^1^ | 51.97(8) |
| O7—Gd1—O2^1^ | 142.04(8) | O1^1^—Gd1—O7^2^ | 144.59(7) |
| O7—Gd1—O4^3^ | 75.67(8) | O3^5^—Gd1—O2^1^ | 68.84(8) |
| O7—Gd1—O7^2^ | 79.19(9) | O3^5^—Gd1—O4^3^ | 135.34(8) |
| O7—Gd1—O6^4^ | 83.38(8) | O3^5^—Gd1—O7^2^ | 69.38(8) |
| O7—Gd1—O5^4^ | 134.91(8) | O3^5^—Gd1—O6^4^ | 138.76(9) |
| O7—Gd1—O1^1^ | 100.36(8) | O3^5^—Gd1—O5^4^ | 140.64(8) |
| O7—Gd1—O3^5^ | 80.05(9) | O3^5^—Gd1—O1^1^ | 75.63(8) |
| O7—Gd1—O8^2^ | 127.44(8) | O3^5^—Gd1—O8^2^ | 88.77(9) |
| O6^4^—Gd1—O2^1^ | 105.77(8) | O8^2^—Gd1—O2^1^ | 74.42(8) |
| O6^4^—Gd1—O7^2^ | 143.00(7) | O8^2^—Gd1—O7^2^ | 49.15(7) |
| O6^4^—Gd1—O1^1^ | 70.61(8) | O8^2^—Gd1—O6^4^ | 130.47(8) |
| O5^4^—Gd1—O2^1^ | 71.80(8) | O8^2^—Gd1—O1^1^ | 126.32(8) |

Symmetry transformations used to generate equivalent atoms: ^1^1+x,1/2-y,1/2+z; ^2^1+x,+y,+z; ^3^2-x,-y,1-z; ^4^2-x,1-y,1-z; ^5^1-x,-y,1-z.
